# Beyond proteolysis: rational modification of mucin-derived peptidomimetics with enhanced metal-mediated antimicrobial activity

**DOI:** 10.1039/d6ra02768g

**Published:** 2026-07-09

**Authors:** Anna Ślusarczyk, Denise Bellotti, Silvia Leveraro, Tomasz Janek, Fabio Zobi, Maurizio Remelli, Joanna Wątły

**Affiliations:** a Faculty of Chemistry, University of Wrocław F. Joliot-Curie 14 Wrocław 50-383 Poland anna.slusarczyk@uwr.edu.pl joanna.watly2@uwr.edu.pl; b Department of Chemical, Pharmaceutical and Agricultural Sciences, University of Ferrara Via Luigi Borsari 46 44121 Ferrara Italy denise.bellotti@unife.it silvia.leveraro@unife.it maurizio.remelli@unife.it; c Department of Biotechnology and Food Microbiology, Wrocław University of Environmental and Life Sciences Chełmońskiego 37 51-630 Wrocław Poland tomasz.janek@upwr.edu.pl; d Department of Chemistry, Fribourg University Chemin Du Musée 9 Fribourg 1700 Switzerland fabio.zobi@unifr.ch

## Abstract

Mucin-derived peptides constitute attractive antimicrobial candidates, but their clinical application is restricted by limited stability and moderate efficacy. To address these limitations, we modified d-amino-acid-containing peptidomimetics and investigated their Cu(ii) and Zn(ii) complexes with respect to coordination chemistry, structure, proteolytic resistance, and antimicrobial activity. Potentiometric, spectroscopic, and DFT studies revealed that metal binding donor sets are analogous to those of the native peptide, producing only minor local conformational effects without significant global structural rearrangement, as confirmed by circular dichroism analysis. In contrast to the modest structural changes, biological activity was strongly influenced by chirality and metal coordination. The fully d-configured analogue displayed the highest antimicrobial potency, particularly at pH 5.5, and its Zn(ii) and Cu(ii) complexes showed enhanced antibacterial and antifungal effects relative to the native system. Proteolytic assays demonstrated rapid plasma degradation of the native peptide and the partially modified analogue, whereas the fully d-substituted peptidomimetic remained largely intact after 2 h. All compounds exhibited minimal hemolytic and cytotoxic effects. These findings demonstrate that d-amino-acid incorporation combined with metal coordination significantly improves both enzymatic stability and antimicrobial performance of mucin-derived peptides.

## Abstract 2

Strategic d-amino-acid modification combined with Cu(ii)/Zn(ii) coordination preserves metal-binding modes while markedly enhancing proteolytic stability and antimicrobial activity of mucin-derived peptidomimetics with minimal toxicity.

## Graphical abstract

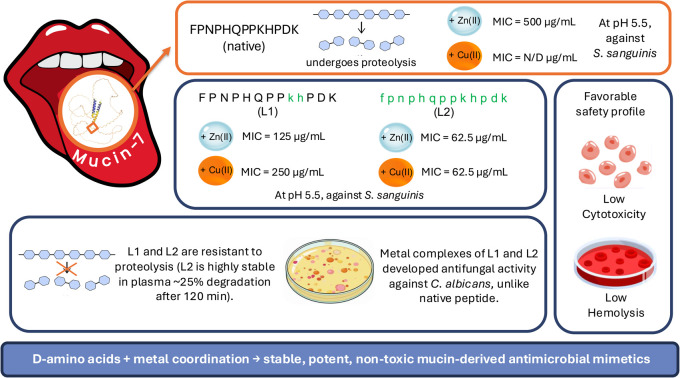

## Introduction

Throughout evolution, bacteria have faced constant threats from competing microbes, bacteriophages, and predators, leading to the development of complex defense systems that ensure survival and confer resistance to antibiotics and other antimicrobial agents.^1,2^ The oral cavity contains hundreds of commensal and potentially pathogenic species which, when homeostasis is disrupted, may threaten human health.^3,4^ Oral homeostasis is primarily maintained by saliva, a fluid continuously secreted by the salivary glands.^5^ Reduced saliva production impairs functions such as taste, mastication, and swallowing, and increases susceptibility to oral diseases (candidiasis, periodontal disease, dental caries) as well as respiratory infections.^6,7^ As a key component of the immune system, saliva possesses extensive protective and healing properties,^8^ largely mediated by its proteins and antimicrobial peptides (AMPs), including mucins, histatins, cystatins, defensins, and cathelicidins, which collectively support oral health.^9,10^

Salivary AMPs are key components of innate immunity, providing broad protection against bacteria and fungi.^11^ Beyond direct antibacterial effects, they have also been reported to exhibit antiviral and immunomodulatory activities.^12^ Owing to their broad-spectrum efficacy and lower tendency to induce resistance, AMPs represent a promising alternative to conventional antibiotics in combating antibiotic resistance.^13,14^

AMPs act through multiple mechanisms, including inhibition of cell wall synthesis, interference with intracellular targets (DNA, proteins, ribosomes), and disruption of microbial membranes *via* electrostatic interactions, leading to permeabilization (barrel-stave, toroidal-pore, carpet models) or detergent-like solubilization.^15^ They also modulate host immunity by influencing cytokine levels, ROS production, and leukocyte differentiation. Importantly, AMP activity is often enhanced by physiologically relevant metal ions such as Cu(ii) and Zn(ii).^14,16^ These ions can (i) limit microbial growth through nutritional immunity and (ii) modify peptide charge and stabilize active conformations,^16,17^ as demonstrated in previously published studies.^18–28^

It should be taken into account that naturally occurring salivary proteins, like most proteins, are rapidly degraded by proteolytic enzymes present in the human body, including those in the oral cavity, gastrointestinal tract, and serum. This degradation can lead to a loss of structural integrity and, consequently, to a reduction or complete loss of antimicrobial activity.^29,30^

**Fig. 1:**
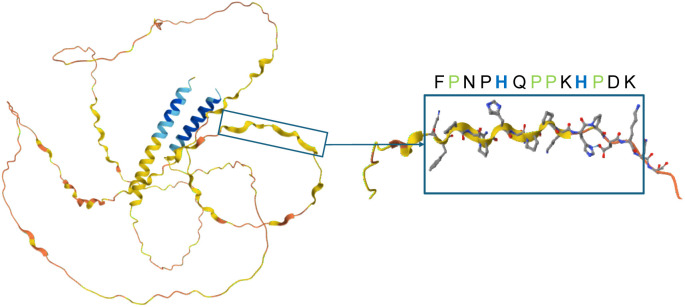
AlphaFold-predicted structure of human MUC7 (UniProtKB: Q8TAX7) with an enlarged view of the FPNPHQPPKHPDK fragment investigated in this study (native sequence).^41,42^

Peptides derived from mucin glycoprotein 2 (MG2, called also mucin 7-MUC7, Fig. 1), a component of saliva that protects the respiratory epithelium, have attracted increasing research interest in recent years.^31–40^

In our previously published study, focusing on the proline rich peptide FPNPHQPPKHPDK (residues 84-96 of MUC7), a naturally occurring fragment generated through proteolytic cleavage of MUC7 glycoprotein, we demonstrated that this peptide is itself prone to further enzymatic degradation, especially at the lysine–histidine (K–H) site. Our findings demonstrated that the trypsin-derived fragments FPNPHQPPK and HPDK differ in their coordination behavior with Cu(ii) and Zn(ii) ions, as well as in their functional properties, particularly regarding metal-binding affinity and antimicrobial activity. Among these, the shortest fragment, HPDK, was found to form the most thermodynamically stable complexes, with both Cu(ii) and Zn(ii) ions and to exhibit superior, pH-dependent antimicrobial activity (MIC = 125 µg mL^−1^ for *Streptococcus sanguinis* PCM 2335 at pH 5.4). These results support the concept of ‘nutritional immunity’ through metal sequestration and suggest that natural proteolytic processing of mucin-derived peptides may enhance their functional potential.^31^

Building on these insights and previous reports demonstrating enhanced antimicrobial activity and improved proteolytic stability of peptidomimetics,^43–47^ we sought to determine whether rational modification of d-amino-acid-containing peptidomimetics could provide superior enzymatic stability and antimicrobial activity compared to the corresponding naturally cleaved fragments.

**Fig. 2:**
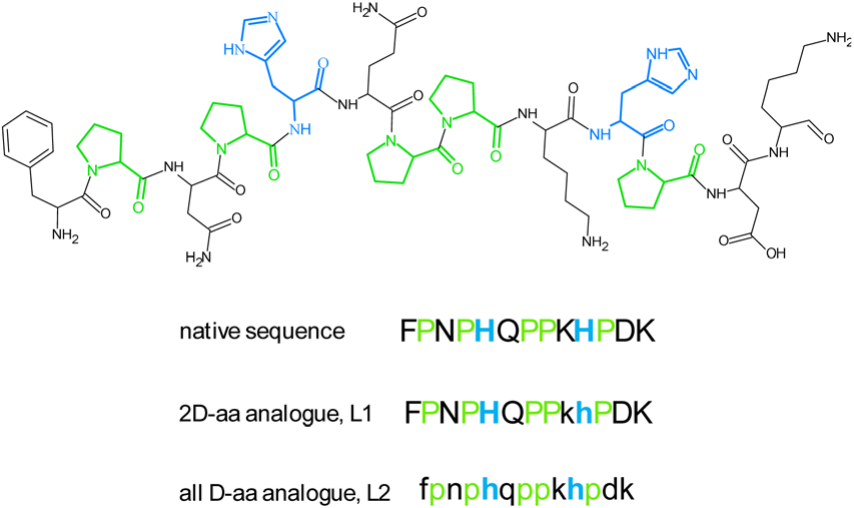
Structural formula and amino acid sequences of the native peptide derived from the naturally occurring salivary protein MUC7 (FPNPHQPPKHPDK) and its two peptidomimetics: FPNPHQPPkhPDK (with two d-amino acid substitutions (2d-aa), L1) and fpnphqppkhpdk (all d-amino acid residues (all d-aa), L2). d-amino acids are indicated in lowercase letters, while naturally occurring l-amino acids are shown in uppercase.

To explore this hypothesis, we have modified two peptidomimetic analogues based on the native FPNPHQPPKHPDK sequence. In the first, d-amino acids were selectively introduced at the trypsin-sensitive cleavage site: lysine (K) and histidine (H), to reduce susceptibility to degradation while preserving the overall sequence framework (Fig. 2A). In the second, a fully d-configured analogue of the peptide was synthesized (Fig. 2B), allowing us to assess the broader impact of stereochemical inversion on peptide stability, structure, and function.

To comprehensively evaluate the impact of these modifications, we pursued six main objectives: (i) to characterize the coordination behavior of Cu(ii) and Zn(ii) ions with the modified analogues; (ii) to analyze their secondary structure and the influence of metal binding on conformational changes; (iii) to compare the thermodynamic stability of the metal complexes; (iv) to investigate how d-amino acid substitution affects antimicrobial activity; (v) to assess the biological stability of the peptidomimetics; (vi) to evaluate cytotoxicity effect on the health human cells. Additionally, we aimed to determine whether such modifications could elicit a more effective antimicrobial response than the native sequence and the fragments generated through its natural proteolysis.

## Experimental

### Experimental procedures

A series of potentiometric titrations was conducted to determine the protonation constants of the ligands as well as the stability constants of their Cu(ii) and Zn(ii) complexes. The coordination behavior of Cu(ii) ions, including the identification of donor atoms (types and number), was elucidated using a combination of spectroscopic techniques, such as: ultraviolet-visible (UV-vis) absorption spectroscopy, circular dichroism (CD), and electron paramagnetic resonance (EPR) spectroscopy. These complementary methods provided detailed insights into the coordination geometry and metal-binding sites.

To support the experimentally derived coordination modes and thermodynamic stability, theoretical calculations were also performed.

The secondary structure of the ligands and their complexes, as well as conformational changes induced upon metal coordination, were evaluated using far-UV CD spectroscopy to further explore structure-function relationships. Biological assays were conducted to assess the antimicrobial activity of the peptidomimetic systems, both in the metal-free form and upon complexation with metal ions. These tests were performed against a panel of clinically relevant oral pathogens, with particular attention paid to pH conditions representative of the oral cavity,^48–51^ which can significantly influence the activity of antimicrobial agents. In addition, hemolytic activity and cytotoxicity toward normal human dermal fibroblasts (NHDFs) were evaluated to assess the biocompatibility and safety profile of the investigated compound.

The biological stability of the peptidomimetics containing d-amino acid residues was examined by monitoring the presence or absence of cleavage products, thereby evaluating their resistance to enzymatic degradation. For this purpose, trypsin digestion assays were performed, as trypsin-like enzymes are naturally present in human saliva.^52–56^ In addition, the resistance to proteolysis of the unmodified peptides was investigated in human plasma. High-performance liquid chromatography (HPLC) was used to monitor peptide degradation over time, enabling estimation of the half-life time of the peptides in human plasma.

### Materials

The analyzed peptidomimetics were based on the MG2 (mucin glycoprotein 2, also known as mucin 7, MUC7) 84-96 sequence fragment (FPNPHQPPKHPDK), with unprotected N- and C-termini: FPNPHQPPkhPDK (L1), and fnpnhqppkhpdk (L2), commercially synthesized by KareBay™ Biochem company with a certified purity of 98% (Fig. S1). Both ligands were analyzed as obtained (without additional purification). Metal ion solutions were prepared from perchlorate hexahydrate salts: Zn(ClO_4_)_2_ and Cu(ClO_4_)_2_ (Sigma-Aldrich) and their concentrations were determined using inductively coupled plasma optical emission spectrometry (ICP-OES). An iCAP 7400 DUO ICP optical emission spectrometer (Thermo Fisher Scientific, Spectro-Lab) was used for this purpose. All samples were prepared using ultra-pure water of the 1st degree of purity according to the ISO 3696:1999 standard (produced by Hydrolab Ultra UV system). Solid substances were weighed using analytical balances: Sartorius R200D and Mettler AE 240 with an accuracy of ±0.00001 g.

### Electrospray ionization mass spectrometry (ESI-MS)

High-resolution electrospray ionization mass spectra (ESI-MS) were recorded using a Bruker Apex Ultra FT-ICR mass spectrometer equipped with an Apollo II ESI source (Bruker Daltonik, Germany). Measurements were conducted in positive ion mode over a mass-to-charge ratio (*m*/*z*) range of 150–2000. The instrumental parameters were as follows: capillary voltage set at 4500 V, nitrogen used as the drying gas at a temperature of 170 °C, and ion energy set at 5 eV. Samples were infused at a flow rate of 3 µL min^−1^. Prior to data acquisition, external calibration was performed using the TuneMix™ mixture (Agilent Technologies, Santa Clara, CA, USA) in quadratic regression mode. Complexes with Zn(ii) and Cu(ii) ions were dissolved in a 1 : 1 (w/w) methanol/water mixture (MeOH, Supelco, HPLC grade), which ensured sufficient sample volatility for efficient electrospray formation. The concentration of peptides was 0.0001 M, and metal–ligand complexes were prepared in a 0.9 : 1 molar ratio (M : L). Data processing and simulation were performed using Bruker Compass DataAnalysis software (version 4.2).^57^

### Potentiometric measurements

Potentiometric measurements were carried out at a constant temperature of 25 °C under an inert argon atmosphere. The stability constants of all ligands and their complexes with Cu(ii) and Zn(ii) ions were calculated based on titration curves recorded within the pH range of 2.0–11.0. Each titration was performed in a total volume of 2.6 mL. The experimental setup consisted of a Metrohm Titrando 905 titrator equipped with a Mettler Toledo InLab Semi-Micro combined pH electrode. The titration was conducted in a thermostated glass cell fitted with a magnetic stirrer and a microburette for precise dispensing of titrant. Ligands were dissolved in a solution of 0.004 M HClO_4_ (prepared from 70–72% perchloric acid, Supelco) with an ionic strength (*I*) adjusted to 0.1 M using NaClO_4_ (Sigma-Aldrich). The ligand concentration was 0.0004 M, and in complexation studies, a metal-to-ligand molar ratio (M : L) of 0.9 : 1 was used. The titrant was a carbonate-free 0.1 M NaOH solution (Supelco), which was potentiometrically standardized using an acidic potassium phthalate solution (*C* = 0.004 M, Sigma-Aldrich).

Prior to each potentiometric titration, the electrode was calibrated using a solution of 0.004 M HClO_4_ with an 0.1 M ionic strength (NaClO_4_). Electrode parameters were assigned using the GLEE software.^58^ Accurate concentrations of all solutions used in the analysis were determined using the HYPERQUAD 2008 program,^59^ which also provided standard deviations automatically. Hydrolysis constants of Cu(ii) and Zn(ii) were sourced from known literature data.^60,61^ The speciation profiles of the metal–ligand systems were calculated with HySS (version 2009),^62^ while all plots and graphical representations of the results were generated using OriginPro.^63^

### Spectroscopic studies

The UV-vis spectroscopic measurements were performed using a Jasco V-750 spectrophotometer. The solutions were placed in quartz cuvettes with an optical path length of 1 cm, and spectra were recorded in the wavelength range of 200–800 nm. Circular dichroism (CD) spectra were obtained under comparable experimental conditions using a Jasco J-1500 spectropolarimeter. Depending on the type of analysis, measurements were recorded either in the far-UV region (190–250 nm) or in the full CD range (225–800 nm), employing quartz cuvettes with path lengths of 0.02 cm (far-UV CD) or 1 cm, respectively. Direct CD signals (*Θ*) were converted to molar ellipticity (Δ*ε*) using the Jasco Spectra Manager software. Ligand concentrations were 0.0004 M, and the metal-to-ligand molar ratio (M : L) was 0.9 : 1. Spectra were acquired at pH values ranging from 3 to 11/11.5, with measurements recorded at 0.5 pH unit intervals for full-range CD and UV-vis analyses, and at 2.0 pH unit intervals for far-UV CD. pH adjustments were made by adding small volumes of NaOH solution (Eurochem BGD). CD spectra were visualized and processed using Jasco Spectra Analysis Software.^64^ All UV-vis and CD measurements were carried out using ligand and complex solutions obtained from prior potentiometric titrations, in which ligands were initially dissolved in aqueous 0.0004 mM HClO_4_ with *I* = 0.1 M NaClO_4_.

Electron paramagnetic resonance (EPR) spectra were recorded at 77 K using a Bruker ELEXSYS E500 CW-EPR spectrometer, operating in the X-band region at a microwave frequency of approximately 9.6 GHz. The instrument was equipped with an NMR teslameter and a frequency counter, and measurements were conducted in the magnetic field range of 2500–3700 G. Ligand solutions were prepared by dissolving the ligands in 1.5 mL of 0.004 M HClO_4_ with 0.1 M ionic strength (NaClO_4_). The final concentrations of the peptide ligand and Cu(ii) ions (Cu(ClO_4_)_2_ × 6H_2_O, Sigma-Aldrich) were 0.0012 M and 0.001 M, respectively. Additionally, 0.5 mL of ethylene glycol (Eurochem BGD) was added to each sample as a cryoprotectant. Measurements were performed at various pH values, adjusted in increments of 1 unit within the pH range 3.0–11.0 by addition of small amounts of concentrated NaOH solution (Eurochem BGD). The exact pH values were determined using a Mettler Toledo SevenDirect SD20 pH meter equipped with a Mettler Toledo InLab Semi-Micro electrode. Experimental spectra were simulated using two programs: DoubletExact (based on the diagonalization of the energy matrix for magnetic field calculations),^65^ and WinEPR SimFonia. The final EPR spectra were visualized and plotted using OriginPro software.^63^

### Computational details

To support the experimental coordination modes and thermodynamic stability, theoretical calculations were performed at the density functional level of theory. All calculations were carried out using the Gaussian 09 software. Geometry optimizations and frequency analyses were conducted using the B3LYP functional,^66,67^ coupled with the 6-31G(d,p) basis set. For both metal ions, the default spin formalism was applied, and default Gaussian 09 values were used for numerical integration grids, SCRF, and geometry optimization convergence criteria. No symmetry constraints were applied during the geometry optimizations. The nature of the stationary points was confirmed by computing vibrational frequencies to ensure they were true minima. All calculations were performed in water with the solvent effects included using the SMD implicit solvation model.^68^

The relative thermodynamic stability of the metal-bound peptide species was assessed based on their calculated relative energies. All energies are given in kJ mol^−1^.

### *In vitro* antimicrobial activity

This study aimed to determine the *in vitro* antimicrobial activity of the tested compounds, expressed as the minimum inhibitory concentration (MIC), a key parameter in evaluating the efficacy of antimicrobial agents. The effectiveness of antimicrobial substances against pathogenic bacteria may vary depending on the pH of the surrounding environment, a factor that was considered during the assessment process. The antimicrobial activity was tested against the following microbial strains: *Escherichia coli*, *Pseudomonas aeruginosa*, *Staphylococcus aureus*, *Streptococcus mutans*, *Streptococcus sanguinis*, *Candida albicans*, and *Enterococcus faecalis*. Four reference strains (*E. coli* ATCC 25922, *P. aeruginosa* ATCC 15442, *E. faecalis* ATCC 29212, and *S. aureus* ATCC 25923) were obtained from the American Type Culture Collection (ATCC) and cultured at 37 °C in Mueller-Hinton Broth (MHB; Merck Millipore, Darmstadt, Germany). The remaining two strains (*S. mutans* PCM 2502, *S. sanguinis* PCM 2335) were obtained from the Polish Collection of Microorganisms (PCM), while *C. albicans* SC5314 was included as the fungal reference strain.^69^*S. mutans* PCM 2502 and *S. sanguinis* PCM 2335 were grown in Brain Heart Infusion (BHI) broth (Merck Millipore, Darmstadt, Germany) under anaerobic conditions (85% N_2_, 10% H_2_, 5% CO_2_) at 37 °C. *C. albicans* was cultured aerobically at 37 °C in Yeast Extract Peptone Dextrose (YPD) broth (A&A Biotechnology, Gdańsk, Poland). MIC values were determined using a two-fold serial dilution method in broth,^70^ carried out in either MES buffer (2-(*N*-Morpholino)ethanesulfonic acid, pH 5.5) or HEPES buffer (4-(2-hydroxyethyl)piperazine-1-ethanesulfonic acid, pH 7.4), both from Merck Millipore (Darmstadt, Germany). Final concentrations of peptides and their metal complexes ranged from 7.8 to 500 µg mL^−1^. To each well, 10 µL of an adjusted microbial suspension (OD_600_ = 0.1), corresponding to approximately 1–5 × 10^7^ colony-forming units (CFU) mL^−1^, was added. Incubation was performed under aerobic (24 h) or anaerobic (72 h) conditions at 37 °C, as appropriate for each strain. Each assay was performed in triplicate. Control experiments assessing the antimicrobial activity of metal ions alone showed no significant effect, thereby confirming the accuracy and reliability of the experimental procedure.

### Hemolytic assay

Sheep red blood cells (SRBCs) were obtained from Pro Animali Company (Wrocław, Poland). The cells were centrifuged at 2500 rpm for 5 min at 10 °C, and the erythrocyte pellet was washed three times with MES buffer (pH 5.5), consistent with the conditions used in the antimicrobial assays. Solutions of the peptides and their metal complexes were prepared in MES buffer to a final concentration of 500 µg mL^−1^. Approximately 2 × 10^7^ SRBCs were suspended in each dilution, incubated at room temperature for 30 min, and subsequently centrifuged to pellet intact erythrocytes. The release of hemoglobin into the supernatant was measured at 540 nm using a Spark® microplate reader (Tecan Trading AG, Switzerland). Sterile MES buffer served as the negative control, while 0.1% Triton X-100 was used as the positive control. The percentage hemolysis for each sample was calculated according to the following formula:
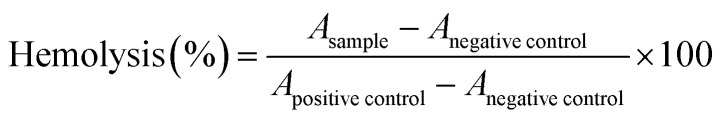
where *A* represents the absorbance at 540 nm. All experiments were performed in triplicate, and mean hemolysis values were reported.

### Cytotoxicity assay

Normal Human Dermal Fibroblasts (NHDFs) were obtained from Lonza (Basel, Switzerland) and maintained in α-Minimum Essential Medium (α-MEM; Institute of Immunology and Experimental Therapy, Wrocław, Poland) supplemented with 10% fetal bovine serum (FBS; Capricorn Scientific, Ebsdorfergrund, Germany), 2 mM glutamine, and antibiotics (100 U mL^−1^ penicillin and 100 µg mL^−1^ streptomycin; Merck Millipore, Darmstadt, Germany). Cells were plated in 96-well plates at a density of 3 × 10^3^ per well in 100 µL of medium and cultured at 37 °C in a 5% CO_2_ atmosphere for 24 h, or until reaching approximately 80% confluence. NHDFs were then treated for 24 h with 500 µg mL^−1^ of the peptides and their Cu(ii) and Zn(ii) complexes (1 : 1 molar ratio). Cell viability and proliferation were evaluated using the standard MTT assay (3-(4,5-dimethylthiazol-2-yl)-2,5-diphenyltetrazolium bromide; Merck Millipore, Darmstadt, Germany), according to the manufacturer's instructions, with absorbance recorded at 570 nm. Cell viability was calculated using the following formula:
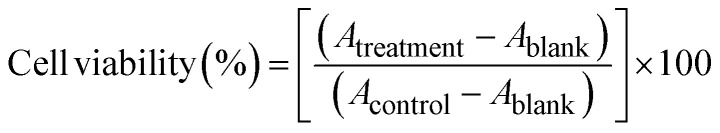
where *A* represents absorbance at 570 nm. Measurements were made in three independent experiments, each performed in triplicate.

## Proteolytic activity

### Trypsin digestion experiment

Peptidomimetics FPNPHQPPkhPDK and fpnphqppkhpdk (1 mg of each) were separately incubated with trypsin (5 mg mL; 100 µL) in ammonium carbonate buffer (100 mM, pH 8.2; 900 µL) for 24 h at 37 °C in a water bath. To terminate the enzymatic reaction, trifluoroacetic acid (fluorochem) was added to reduce the pH to approximately 3.5 (TFA final concentration was about 5%). The resulting mixture was then transferred to Vivaspin 500 centrifugal concentrators (PES membrane, 5000 Da molecular weight cut-off), and subsequently analyzed by matrix-assisted laser desorption/ionization time-of-flight mass spectrometry (MALDI-TOF MS) using a JEOL JMS-S3000 SpiralTOF -plus Ultra-High Mass Resolution system operated in positive ion mode (*z* = +1). As a control, a sample containing only trypsin and buffer (without peptide) was prepared and analyzed under identical conditions. Sinapic acid was used as the MALDI matrix, and was prepared by dissolving 10 mg of sinapic acid in 1 mL of a 50 : 50 (w/w) acetonitrile/water solution containing 0.1% TFA. The sample and matrix solutions were mixed in a 1 : 1 (v/v) ratio and spotted onto a 96-well stainless steel MALDI target plate, followed by air-drying at room temperature. Mass spectra were processed and interpreted using msTornado Analysis software (version 2.0.11.1).

### Resistance to degradation in human plasma

The persistence of the peptidomimetics in human plasma was assessed using high-performance liquid chromatography (HPLC). The experiments were conducted in human plasma obtained from a single donor, as confirmed by an anonymized sample code. The plasma originated from a pooled collection of 40 healthy donors at the University Hospital in Ferrara, Italy. Informed consent was obtained from all participants and/or their legal guardians before blood donation. Peptide samples were prepared by dissolving the compounds in 1 mL of 0.01 M ammonium acetate buffer (Fluka), pH 7.4, and 1 mL of human plasma to a final concentration of 0.0001 M. l-phenylalaninol (Sigma Chemical) was added as internal standard. Each peptide sample is then incubated at 37 °C. At regular time intervals (0, 5, 10, 15, 30, 45, 60, 90 and 120 minutes), 200 µL aliquots were taken from the incubated solution and enzymatic reactions were blocked by adding 300 µL of 0.5 M HClO_4_, and then, the sample is centrifuged for 6 minutes at 14.000 rpm. The supernatant was then filtered and analyzed by HPLC. The chromatographic analysis was performed using a PerkinElmer Series 200 HPLC system equipped with a UV detector from the same manufacturer. Detection was carried out at a wavelength of 210 nm and a temperature of 298 K. The used analytical column was an Agilent Poroshell 120 SB-C18 (4.6 × 100 mm, 2.7 µm particle size, 120 Å pore size). The mobile phase consisted of a mixture of HPLC-grade acetonitrile (CARLO ERBA) and water (13 : 87, v/v) containing 0.1% TFA (Fluorochem). Each peptide was analyzed in at least three independent experiments. The cumulative results were processed using OriginPro software, with standard deviations calculated for each data point.^63^

## Results and discussion

### Deprotonation constants

Potentiometric titrations revealed the presence of seven deprotonation constants for each peptidomimetic, as summarized in Table 1. The determined p*K*_a_ values are highly similar for both ligands, and for the native peptide,^31^ and they are consistent with those reported in the literature for both l- and d-amino acids.^48–53^ The distribution diagrams of the protonated species of peptidomimetics as a function of pH, in the range of 2.0–11.0, are presented in Fig. S2.

Deprotonation constants (p*K*_a_) for L1 and L2 peptidomimetics with stability constants (log *β*) for their complexes with Cu(ii) and Zn(ii) ions in aqueous solution of 0.004 M HClO4 with *I* = 0.1 M (NaClO_4_) at 298 K. [L] = 0.0004 M; molar ratio M : L – 0.9 : 1 the standard deviations are reported in parentheses as uncertainties on the last significant figure. C-t and N-t correspond to C-terminus and N-terminus, respectively. Lowercase letters indicate d-amino acids, whereas uppercase letters indicate l-amino acids

**Table:** 

Ligands						
Species	L1 (FPNPHQPPkhPDK)			L2 (fpnphqppkhpdk)		
	log *β*_*jk*_^a^	p*K*_a_^b^	Residue	log *β*_*jk*_^a^	p*K*_a_^b^	Residue
[H_7_L]^5+^	47.38(8)	2.72	C-t	48.44(1)	2.99	C-t
[H_6_L]^4+^	44.66(8)	3.75	Asp	45.45(1)	3.94	Asp
[H_5_L]^3+^	40.91(8)	5.83	His	41.51(1)	6.08	His
[H_4_L]^2+^	35.09(7)	6.64	His	35.43(1)	6.62	His
[H_3_L]^+^	28.45(8)	7.41	N-t	28.81(1)	7.47	N-t
[H_2_L]	21.04(7)	10.17	Lys	21.34(1)	10.09	Lys
[HL]^−^	10.87(10)	10.87	Lys	11.25(1)	11.25	Lys

a Constants are presented as cumulative log *β*_*jk*_ values. *β*(*H*_*j*_*L*_*k*_) = [*H*_*j*_*L*_*k*_]/([H]^*j*^[L]^*k*^), in which [L] is the concentration of the fully deprotonated peptide.

b p*K*_a_ values of the peptides were derived from cumulative constants: p*K*_a_ = log *β*(*H*_*j*_*L*_*k*_) – log *β*(*H*_*j*−1_*L*_*k*_).

c Cu(ii) and.

d Zn(ii) stability constants are presented as cumulative log *β*_*ijk*_ values. *L* stands for a fully deprotonated peptide ligand that binds Cu(ii)/Zn(ii) ion: *β*(*M*_*i*_*H*_*j*_*L*_*k*_) = [*M*_*i*_*H*_*j*_*L*_*k*_]/([M]^*i*^[H]^*j*^[L]^*k*^), where [L] is the concentration of the fully deprotonated peptide. p*K*_a_ = log *β* (*M*_*i*_*H*_*j*_*L*_*k*_) – log *β*(*M*_*i*_*H*_*j*−1_*L*_*k*_).

**Table:** 

Cu(ii) complexes				
Species	cCu(ii)-L1 (FPNPHQPPkhPDK)		cCu(ii)-L2 (fpnphqppkhpdk)	
	log *β*_*jk*_^a^	p*K*_a_^b^	log *β*_*jk*_^a^	p*K*_a_^b^
[CuH_3_L]^3+^	34.75(3)	—	35.1(1)	—
[CuH_2_L]^2+^	28.61(4)	6.14	28.97(2)	6.13
[CuHL]^+^	19.93(7)	8.68	20.84(2)	8.13
[CuL]	11.01(5)	8.92	12.3(2)	8.54
[CuH_−1_L]^−^	1.08(6)	9.93	2.36(2)	9.94
[CuH_−2_L]^2−^	−9.61(6)	10.69	−8.46(2)	10.82

**Table:** 

Zn(ii) complexes				
Species	dZn(ii) - L1 (FPNPHQPPkhPDK)		dZn(ii)-L2 (fpnphqppkhpdk)	
	log *β*_*jk*_^a^	p*K*_a_^b^	log *β*_*jk*_^a^	p*K*_a_^b^
[ZnH_3_L]^3+^	—	—	32.01(3)	—
[ZnH_2_L]^2+^	25.38(1)	—	25.46(1)	6.55
[ZnHL]^+^	—	—	—	—
[ZnL]	8.52(1)	—	8.33(1)	—
[ZnH_−1_L]^−^	−0.77(1)	9.29	−1.75(3)	10.08
[ZnH_−2_L]^2−^	−11.12(1)	10.35	−12.21(2)	10.46

### Cu(ii) complexes

#### Electrospray mass spectrometry (ESI-MS)

Electrospray mass spectrometry (ESI-MS) facilitated the confirmation of the stoichiometry of the complexes formed between the studied peptidomimetics and Cu(ii) ions. Analysis of the obtained spectra, including isotopic distribution, confirmed the exclusive formation of equimolar (1 : 1, ligand-to-metal) complexes and excluded the presence of polynuclear species. The experimental spectra recorded across the entire *m*/*z* range are presented in Fig. S3A and S4A, and all results are summarized in Table S1. The strong agreement between the experimental and simulated *m*/*z* values and isotopic distributions of the Cu(ii)-containing complexes, further validates the accuracy of the proposed interpretations. For the Cu(ii)-L1 and Cu(ii)-L2 complexes, the signal with the highest intensity corresponds to the [CuL]^3+^ complex with an *m*/*z* value of 534.421, whereas for [CuL]^2+^ the *m*/*z* is 801.127. Other signals detected in the ESI-MS spectra are mainly attributed to alkali metal and counter-ion adducts of the Cu(ii) complexes (*e.g.* for the Cu(ii)-L1 system sodium and potassium adduct species were detected, including [CuL + Na^+^]^2+^, [CuL + Na^+^]^3+^, and [CuL + K^+^]^3+^, and in the case of Cu(ii)-L2 system-K^+^, Na^+^, and ClO_4_^−^ adducts were observed, such as [CuL + K^+^]^2+^, [CuL + ClO_4_^−^]^2+^, [CuL + ClO_4_^−^ + K^+^]^2+^, [CuL + ClO_4_^−^]^3+^, [CuL + ClO_4_^−^ + K^+^ + Na^+^]^3+^, and [CuL + Na^+^]^4+^).

#### Potentiometric titrations

**Fig. 3:**
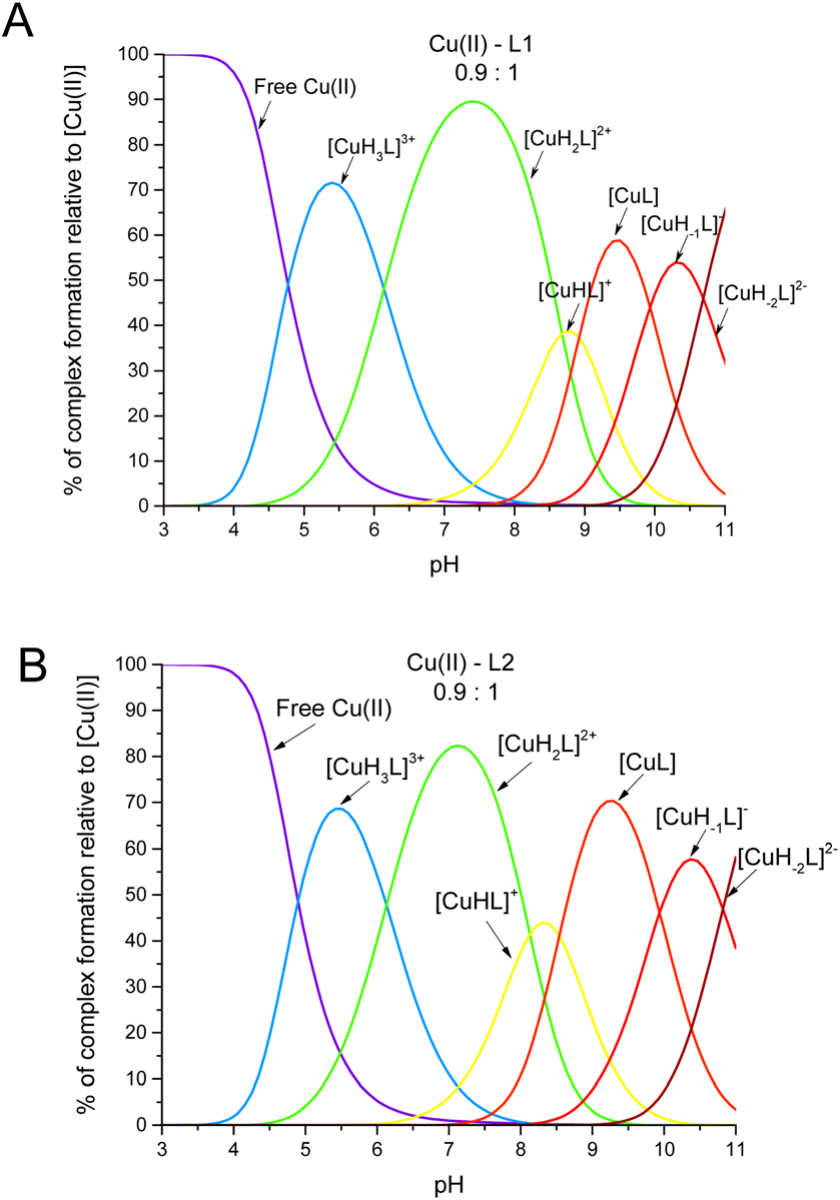
Representative distribution diagram for (A) Cu(ii)-L1 (FPNPHQPPkhPDK); (B) Cu(ii)-L2 (fpnphqppkhpdk) systems in aqueous solution of 0.004 M HClO_4_ with ionic strength *I* = 0.1 M (NaClO_4_). [L] = 0.0004 M; molar ratio M : L – 0.9 : 1. *T* = 298 K.

Potentiometric titrations enabled the identification of six distinct equimolar Cu(ii) complex species for each peptidomimetic within the pH range of 3.0 to 11.0 (Table 1 and Fig. 3). Their maximum concentration occurs at similar pH values, differing by 0.25 pH unit or less, with the exception of the [CuHL]^+^ species, for which the maxima are slightly more separated – at pH 8.8 (Cu(ii)-L1), and at 8.3 (Cu(ii)-L2). The peptides and their complexes with Cu(ii) ions exhibited high solubility, and no turbidity or precipitation was observed across the entire pH range studied. Characterization of the coordination environment of Cu(ii) ions in each of the identified complex species was carried out based on potentiometric and spectroscopic results.

### Cu(ii)-L1 system

**Fig. 4:**
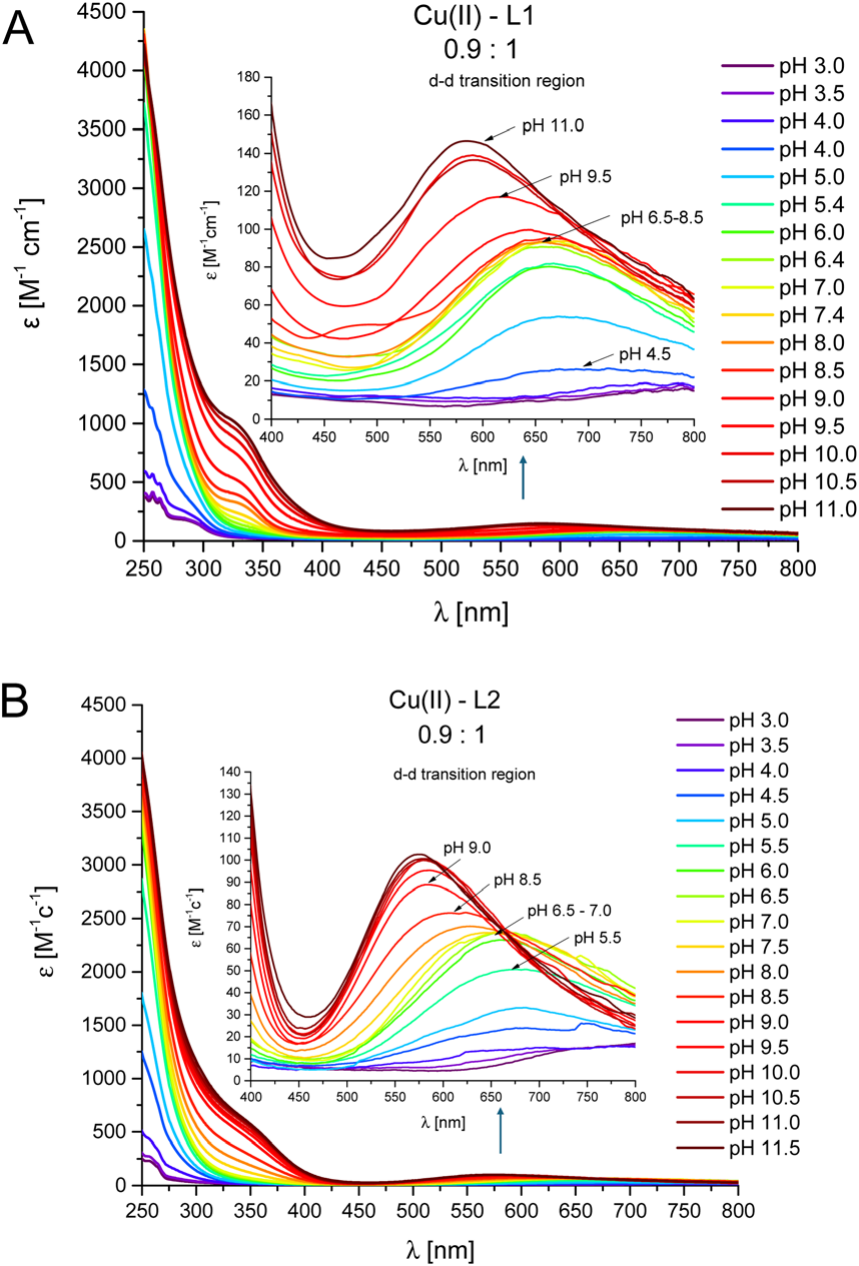
UV-vis spectra for (A) Cu(ii)-L1 (FPNPHQPPkhPDK) and (B) Cu(ii)-L2 (fpnphqppkhpdk) complexes in aqueous solution of 0.004 M HClO_4_ with ionic strength *I* = 0.1 M (NaClO_4_). [L] = 0.00035 M; molar ratio M : L – 0.9 : 1; *T* = 298 K; optical path length = 1 cm.

**Fig. 5:**
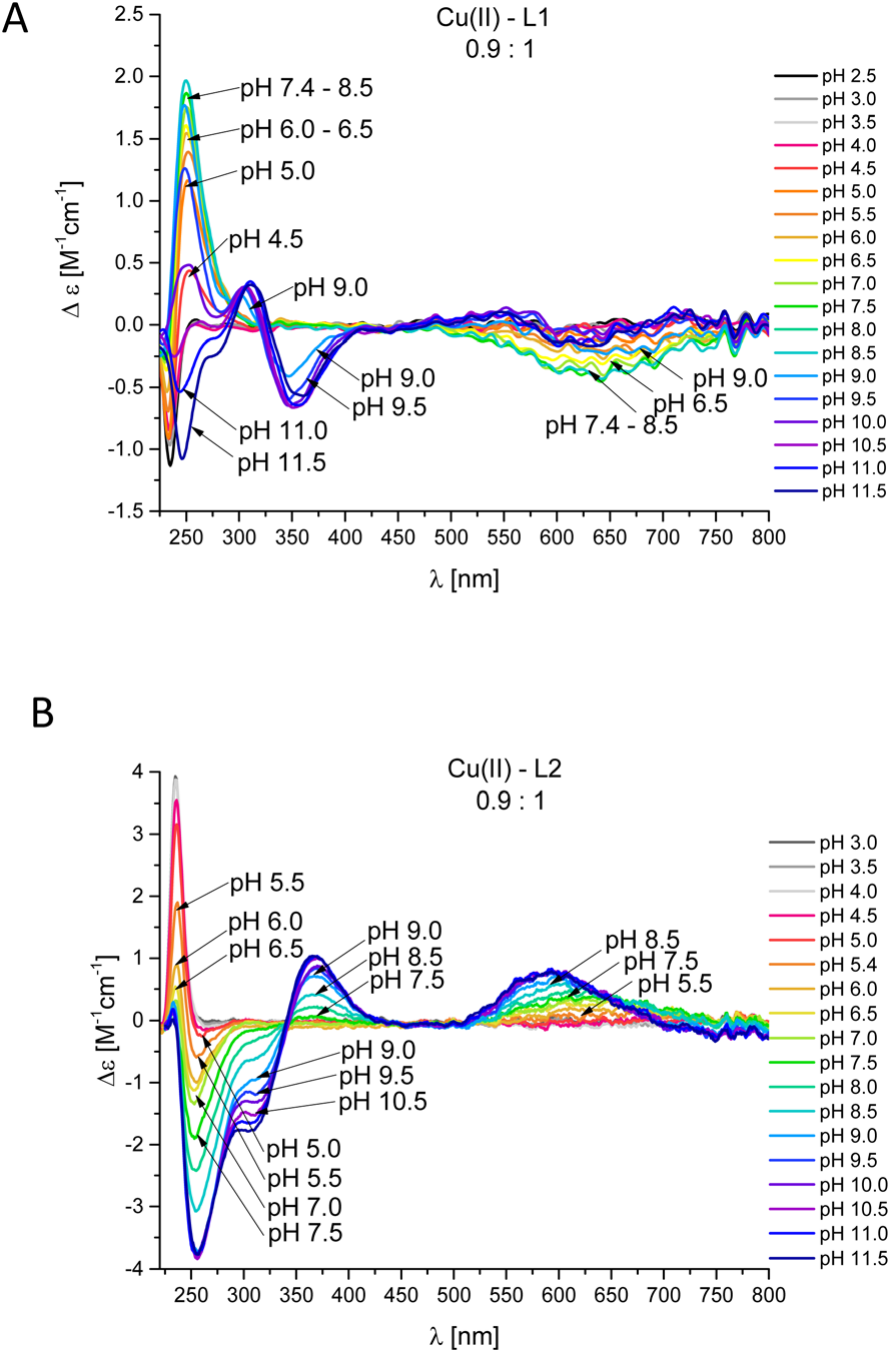
CD spectra for (A) Cu(ii)-L1 (FPNPHQPPkhPDK) and (B) Cu(ii)-L2 (fpnphqppkhpdk) complexes in aqueous solution of 0.004 M HClO_4_ with ionic strength *I* = 0.1 M (NaClO_4_). [L] = 0.00035 M; molar ratio M : L – 0.9:1; *T* = 298 K; optical path length = 1 cm.

The first complex species present solution is [CuH_3_L]^3+^, reaching its maximum concentration at pH 5.5. It is formed through the coordination of two nitrogen atoms, as suggested by by the d–d transition band with a maximum absorption at 667 nm (Fig. 4A).^71,72^ The bands in the CD spectra at: (i) 232 nm (negative Cotton effect), (ii) 252 nm (positive Cotton effect) and (iii) 665 nm (positive Cotton effect) shown in Fig. 5A strongly support the involvement of the imidazole nitrogen from the His residue^73–75^ and the amide nitrogen^76,77^ from the peptide bond, respectively, which is consistent with a 2N coordination mode containing a {1N_im_, 1N_am_} donor set. Additionally, EPR spectroscopy, with parameters: *g*‖ = 2.28 and *A*‖ = 161.8 (Fig. S5A) confirms the coordination of Cu(ii) by two nitrogens.

**Fig. 6:**
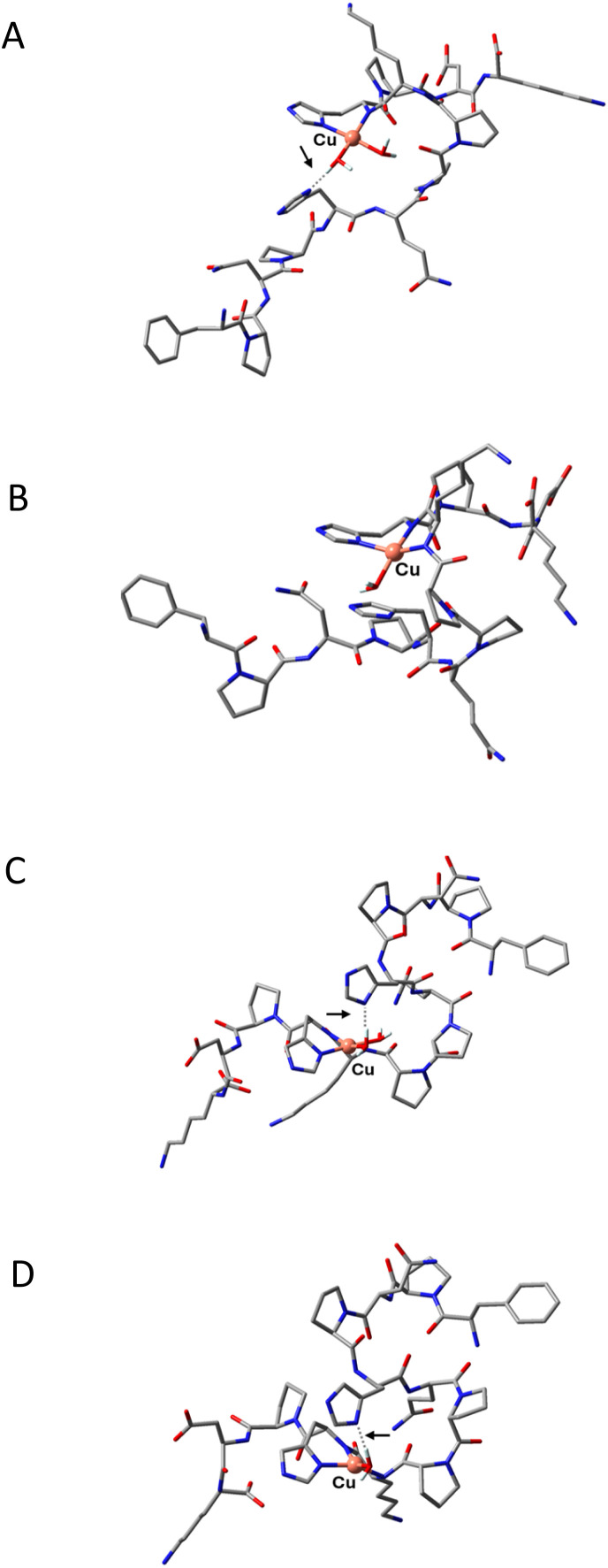
DFT calculated structures for Cu(ii)-L1 (FPNPHQPPkhPDK): (A) [CuH_2_L]^2+^and (B) [CuL] complex species, and for Cu(ii)-L2 (fpnphqppkhpdk): (C) [CuH_2_L]^2+^and (D) [CuL] complex species. Arrows indicate intramolecular H-bonds.

The presence of the next complex species, [CuH_2_L]^2+^, which predominates over the pH range 6.0–8.0, including physiological conditions and the typical pH of saliva,^48–51^ is assigned to the deprotonation of the non-coordinating histidine residue, as indicated by the similar p*K*_a_ values in the ligand and complex: 6.64 and 6.14, respectively (Table 1). Additionally, the absence of significant changes in the spectroscopic parameters confirms that the coordination mode remains unchanged, involving the same {1N_im_, 1N_am_} donor set. DFT calculations further support this model, showing that the Cu(ii) ion is coordinated by the side-chain imidazole nitrogen of d-His and the adjacent deprotonated amide nitrogen. The optimized structures also reveal an intramolecular hydrogen bond between a coordinated water molecule and the imidazole ring of a second His residue (Fig. 6A).

No significant variations in the UV-vis and EPR spectra are observed at the pH values corresponding to the formation of the [CuHL]^+^ species, indicating that this process involves exclusively the non-coordinating deprotonation of the N-terminal –NH_3_^+^ group. The increase in the apparent p*K*_a_ of the N-terminus (8.68 in the complex *vs.* 7.41 in the ligand) occurs after the establishment of the {1N_im_, 1N_am_} coordination mode and likely reflects the altered electrostatic environment and conformational constraints imposed by this binding motif. Such coupled coordination – protonation equilibria are commonly observed in Cu(ii)-peptide systems.^78,79^

Above pH 9.0, a blue shift of the absorption maximum is observed in the UV-vis spectra, shifting from 655 nm at pH 8.5 to 613–590 nm in the pH range 9.5–12 (Fig. 4). This behavior is accompanied by the appearance of new signals in the EPR and CD spectra. At pH 10, two sets of EPR parameters are detected, with *A*‖ values of 163.8 and 171.9 and *g*‖ values of 2.26 and 2.23 (Fig. S5A), indicating the coexistence of species with 2N and 3N coordination modes.^80^ Consistent changes in CD spectra are also observed above pH 9.0, including (i) a gradual decrease in the intensity of the bands at 250 and 650 nm and (ii) the emergence of new bands: with positive Cotton effect at approximately 300 nm and with negative Cotton effect at approximately 350 nm (Fig. 5A). These spectral changes are consistent with the involvement of an additional deprotonated amide nitrogen, leading to a reorganization of ligand-to-metal charge–transfer transitions rather than the emergence of a new type of donor.^81^

Accordingly, in the [CuL] species dominant at pH 9.45, the Cu(ii) ion is most likely coordinated by a {1N_im_, 2N_am_} donor set. This assignment is further supported by DFT calculations, which confirm the presence of this type of coordination mode at higher pH values (9.0–10.5), involving the second amide donor from the d-lysine residue (Fig. 6B).

The subsequent complex species present in solution, [CuH_−1_L]^−^ and [CuH_−2_L]^2−^, arise from nonbinding deprotonation of Lys residues. This is supported by comparable p*K*_a_ values for the free ligand (10.17 and 10.87) and the corresponding complex species (9.93 and 10.69), Table 1. The EPR and UV-vis spectral parameters (Fig. 3A and S5A) further confirm the persistence of the 3N coordination mode, described by a {1N_im_, 2N_am_} donor set.

### Cu(ii)-L2 system

For the Cu(ii)-L2 system, the p*K*_a_ values obtained for the ligand and complex species, the distribution diagrams, and the spectroscopic results are very similar to those observed for the Cu(ii)-L1 system (Table 1, Fig. 3B and4B). This similarity suggests the same coordination modes in both systems, particularly for the [CuH_3_L]^3+^, [CuH_2_L]^2+^, [CuHL]^+^ complex species, which are characterized by a {1N_im_, 1N_am_} donor set. However, subtle differences in the UV-vis spectra and pronounced differences in the CD spectra suggest that the coordination environment, especially above pH 7, where the [CuL] species becomes dominant (Fig. 3B), is not identical. With increasing pH, a clear blue shift of the absorption maximum is observed, from 665 nm at pH 7.0 to 583 nm at pH 9.0, suggesting the coordination of an additional nitrogen donor and a transition to a 3N coordination mode. It is worth noting that the broadening of the UV-vis band at pH 8.5 (Fig. 4B), together with the presence of two sets of EPR parameters at pH 9.0 (Fig. S5B), indicates the coexistence of species with 2N coordination (a residual contribution from the earlier form) and 3N coordination, in agreement with the speciation diagrams (Fig. 3). These findings suggest that coordination of the second amide nitrogen to Cu(ii) occurs at a pH approximately one unit lower than in the Cu(ii)-L1 system, which is further supported by the appearance of new bands in the CD spectra at approximately 300 and 350 nm above pH 8.0 (for the Cu(ii)-L1 system, such bands emerge above pH 9.0, Fig. 5). Nevertheless, in both systems, the [CuL] complex species is characterized by a {1N_im_, 2N_am_} donor set, as further supported by DFT calculations (Fig. 6B and D). The differences observed in the CD spectra relative to the Cu(ii)-L1 complexes are also likely influenced by the contribution of d-amino acids, as reflected in both band intensities and Cotton-effect signs. In the case of the L1-based system, lower band intensities and opposite Cotton-effect signs compared to the L2-based system were observed, which can be attributed to the higher proportion of l-amino acids (only two d-amino acids) compared to the fully d-amino-acid sequence of the L2 peptidomimetic, resulting in optical rotation of circularly polarized light in the opposite direction.^82^ Taken together, these differences in the CD spectra are most likely associated with variations in the chiral environment of the complexes, rather than with changes in the donor atoms involved in Cu(ii) coordination (Fig. 5).

**Fig. 7:**
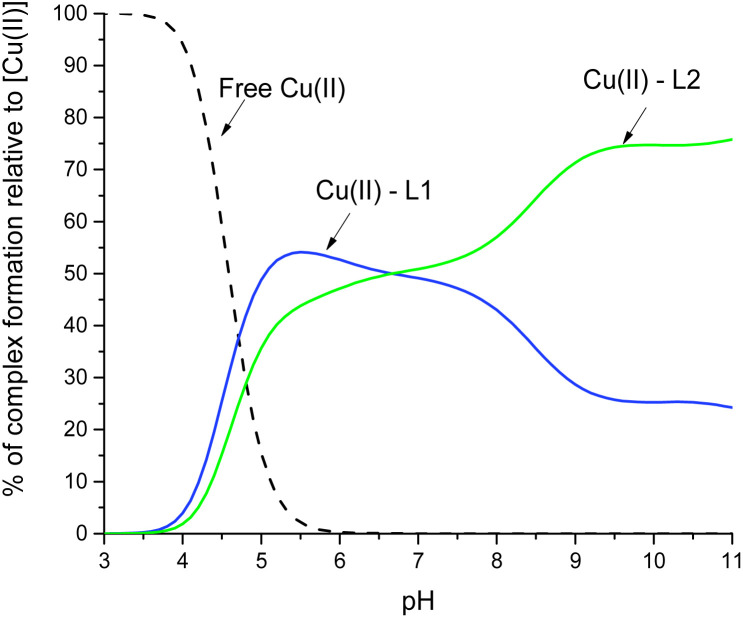
Competition plot between L1 (FPNPHQPPkhPDK), L2 (fpnphqppkhpdk), and Cu(ii) ions describing complex formation at different pH values in a hypothetical situation, in which equimolar amounts of all the reagents are mixed. [Cu(ii)] = [L1] = [L2] = 0.001 M.

A comparison of these two systems using a so-called competitive plot (a theoretical model assuming equimolar concentrations of all reagents and allowing direct comparison of complex formation efficiency as a function of pH, Fig. 7) clearly supports the coordination modes proposed above, as well as the influence of the presence of d-amino acids either (i) throughout the entire peptide sequence or (ii) exclusively at the site most susceptible to enzymatic degradation. This effect is most pronounced above pH 8.0, where the L2 peptide forms more stable complexes and begins to bind the Cu(ii) ion through the second amide nitrogen, whereas in the case of the L1 peptide this process occurs above pH 9.0. In addition, the spatial arrangement of l- and d-amino acid residues in the L1 peptide results in a less favorable preorganization of the system, which in turn leads to reduced affinity toward Cu(ii) ions.

Moreover, the native peptide shows an affinity for metal binding similar to that of the L2 peptide (Fig. S6), which supports that replacement of all l-amino acids with their enantiomers does not result in significant differences in the coordination mode or stability of the Cu(ii) complexes. In contrast, replacing only two key amino acids at internal positions within the peptide sequence leads to observable changes in the coordination behavior and stability of the Cu(ii) complexes at alkaline pH.

DFT data support the experimental findings and indicate a common coordination motif for all three peptides at physiological pH. At pH 7.5–8.0, the computed aqueous energies of the Cu adducts are very similar for all three peptides (*ca.* 3–5 kJ mol^−1^), with the Cu(ii)-L2 adduct calculated to be the most stable system. At higher pH values (9.0–10.5), DFT calculations indicate a thermodynamically preferred energy minimum corresponding to deprotonation and the formation of a {1N_im_, 2N_am_} coordination mode in all three systems, in which the second amide donor is provided by the d-lysine residue (Fig. 6 and S7).

### Zn(ii) complexes

Mass spectrometry (ESI-MS) confirmed that the studied peptidomimetics form only 1 : 1 complexes with Zn(ii) ions, with no evidence of polynuclear species or bis-complexes. In the spectra of the Zn(ii)-L1 and Zn(ii)-L2 systems, the predominant ions are assigned to the [ZnL]^2+^, [ZnL]^3+^, and [ZnL]^4+^ complex species, detected at average *m*/*z* values of 802.059, 535.042, and 401.533 for L1 system, and 802.043, 534.232 and 401.525 for L2-Zn(ii) complex respectively. In addition, for the Zn(ii)-L2 complex, a minor signal assigned to a perchlorate adduct, [ZnL + ClO_4_^−^]^3+^, was observed. A strong agreement between experimental and simulated *m*/*z* values and isotopic patterns validates these findings (Table S1, Fig. S3B and S4B). This stoichiometry was further supported by potentiometric data (Table 1).

**Fig. 8:**
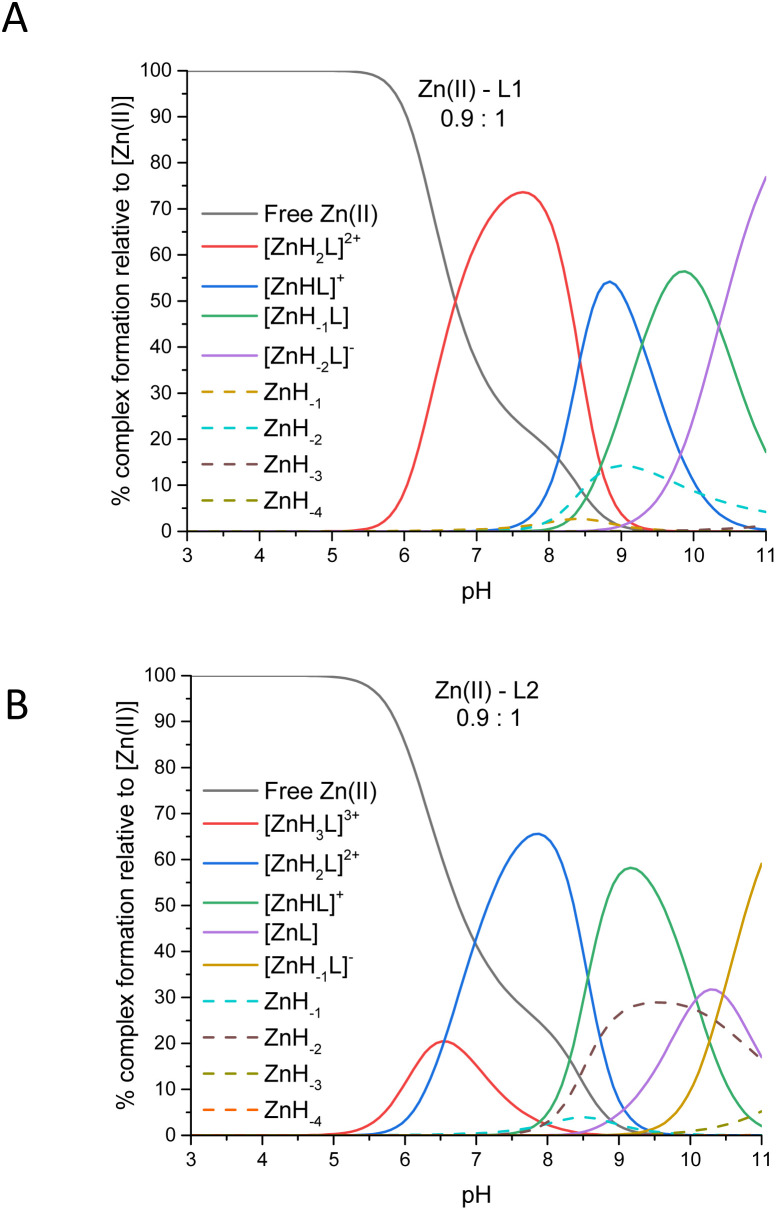
Representative distribution diagram including Zn(ii) ion hydrolysis^60^ (dashed lines) for (A) Zn(ii)-L1 (FPNPHQPPkhPDK); (B) Zn(ii)-L2 (fpnphqppkhpdk) systems in aqueous solution of 0.004 M HClO_4_ with ionic strength *I* = 0.1 M (NaClO_4_). [L] = 0.0004 M; molar ratio M : L – 0.9 : 1; *T* = 298 K.

The formation of zinc complexes begins at a higher pH value than copper complexes – L1 starts to bind Zn(ii) at around pH 5.4 (Fig. 8A), whereas L2 begins to bind Zn(ii) at approximately pH 5.0 (Fig. 8B).

For the Zn(ii)-L1 system, four complex species were identified, whereas five species were detected for the Zn(ii)-L2 system. In both cases, the [ZnH_2_L]^2+^ species is dominant at pH values corresponding to oral cavity conditions.

**Fig. 9:**
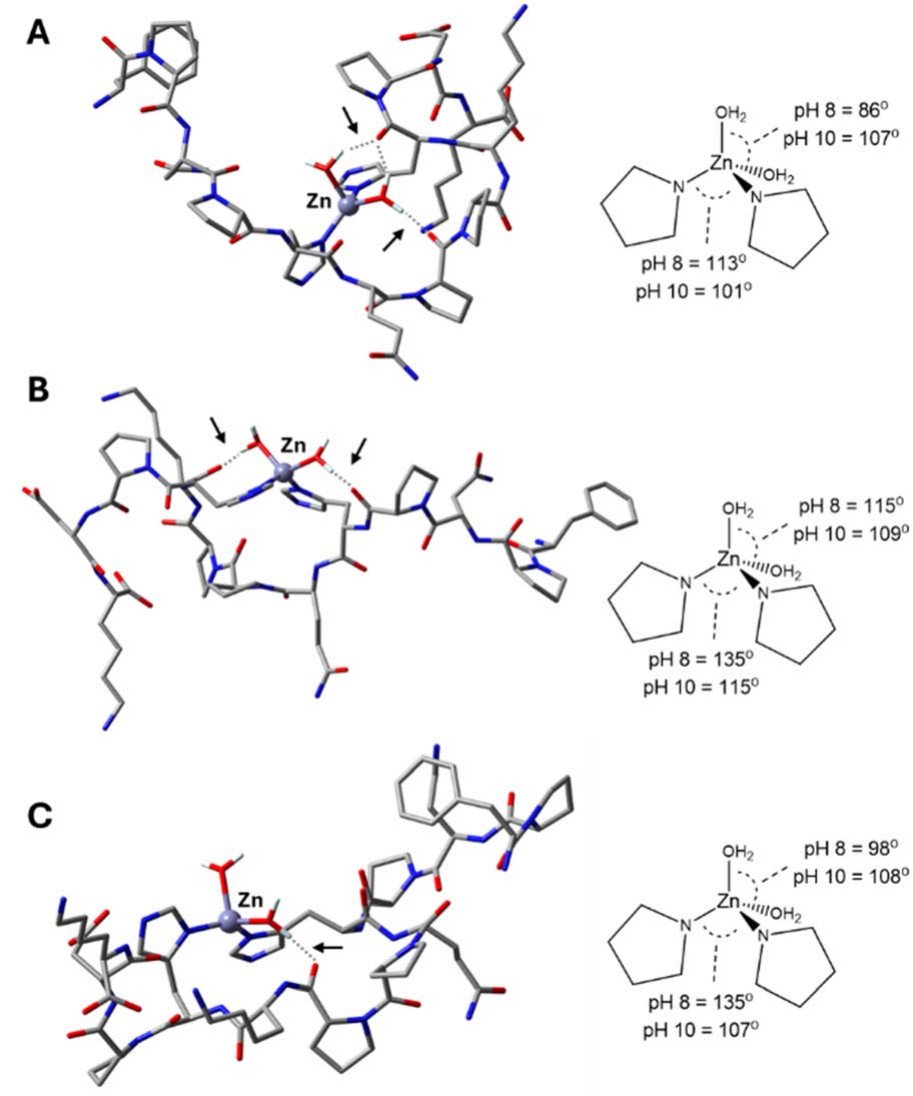
DFT calculation structures for the zinc(ii) complexes with (A) native peptide (FPNPHQPPKHPDK); (B) L1 (FPNPHQPPkhPDK); and (C) L2 (fpnphqppkhpdk) ligands at pH 8 and pH 10. Arrows indicate intramolecular H-bonds.

DFT calculations performed for the native peptide system excluded coordination of the N-terminus to Zn(ii) *via* a {1N_im_, –NH_2_} donor set.^31^ This binding mode was found to be energetically unfavourable due to the rigidity of the peptide imposed by the presence of multiple proline residues (structures involving this donor set did not converge during geometry optimization, indicating that this coordination mode lies high on the potential energy surface).^31^ In addition, DFT studies of Zn(ii) complexes with the L1 and L2 peptidomimetics confirmed a {2N_im_} donor set for all systems in the pH range of 8.0–10.0 (Fig. 9). For the [ZnH_2_L]^2+^ complex species, deprotonation of one of the coordinated water molecules is observed in the potentiometric study (p*K*_a_ = 6.55) for the Zn(ii)-L2 system, which is comparable to the value reported for the native form.^31^

Formation of the [ZnL] species in both systems proceeds *via* a two-step deprotonation process, involving (i) the N-terminus (non-binding deprotonation) and (ii) a second water molecule in the metal coordination sphere. Further complex species arise from noncoordinating deprotonation of lysine residues, as supported by comparable p*K*_a_ values for the free ligands and the corresponding complexes (Table 1).

**Fig. 10:**
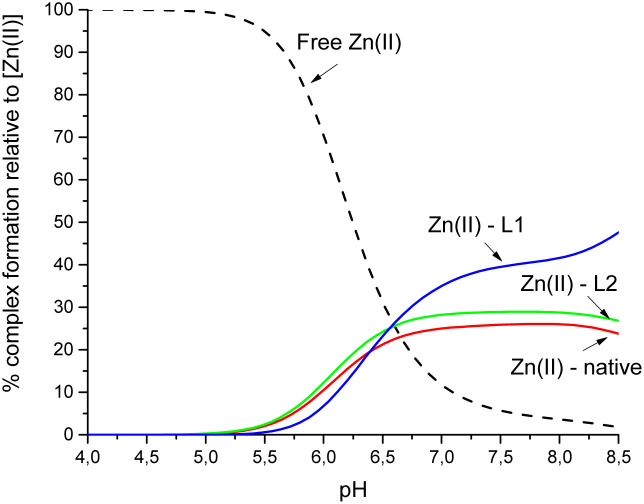
Competition plot between native (FPNPHQPPKHPDK), L1 (FPNPHQPPkhPDK), L2 (fpnphqppkhpdk) peptides/peptidomimetics with Zn(ii), describing complex formation at different pH values in a hypothetical situation, in which equimolar amounts of all the reagents are mixed. Conditions: *T* = 298 K, [Zn(ii)] = [L1] = [L2] = 0.001 M. The plot is shown up to pH 8.5; above pH 8.5, species resulting from zinc hydrolysis (ZnH_−2_) become significant, particularly in the Zn(ii)-L1 system.

Interestingly, although the competition plot (Fig. 10) shows very similar overall Zn(ii) affinities for the native peptide and its d-amino-acid analogue (L2), the L1 ligand forms more stable Zn(ii) complexes. This observation was supported by DFT calculation.

Among the three {2N_im_}Zn(H_2_O)_2_ systems at pH 7.5–8.0, the geometric parameters and relative DFT energies indicate a higher intrinsic stability of the Zn(ii)-L1 complex in aqueous solution. In the L1 peptide, the calculated Zn–ligand bond distances are highly symmetric (Zn(ii)–N_im_ ≈ 2.01 Å and Zn–OH_2_ ≈ 2.04 Å), and the coordination geometry is closer to an ideal tetrahedral arrangement than in the native and L2 peptide adducts. This is consistent with computed relative free energies in water, which show that Zn(ii) forms the most stable adduct with L1, while complexes with the native and L2 peptides are higher in energy by approximately 37 and 51 kJ mol^−1^, respectively.

The DFT results are in good agreement with the experimental competition experiments, which identify the Zn(ii)-L1 complex as the dominant species in this pH range (≈42%), while L2 and the native peptide contribute to a similar extent (≈30% and 28%). The slightly higher population of L2 relative to the native peptide, despite the opposite energetic trend predicted by DFT, likely reflects the influence of intramolecular hydrogen bonding and solvent exposure of the coordinated water molecules (native peptide forms three intramolecular hydrogen bonds and features a coordinated Zn(ii) buried deeper into the peptide, whereas L1 and L2 form only two and one intramolecular hydrogen bonds respectively, with both coordinated waters more exposed to bulk solvent, Fig. 9).

Overall, the calculations indicate that the native peptide accommodates Zn(ii) binding by forming a more highly distorted complex, but the same is increasingly relaxed by coordination to L1 and L2. The computational data suggest that the intrinsic metal–ligand coordination geometry, subtle solvation and secondary-sphere hydrogen-bonding interactions all contribute to the relative stabilization of the Zn(ii)-peptide complexes in solution, favoring the less hindered metal adduct formed with L1. Thus, overall, both computational and experimental data consistently identify L1 as the most stable Zn(ii) complex under the studied conditions. At increasing pH values the relative trend described above persists, with the Zn(ii)-L1 complex being more stable than the native and L2 peptide adducts by *ca.* 20 kJ mol^−1^ but we could not computationally identify significant structural changes that may explain the experimentally observed decrease in stability of the zinc(ii) complexes with native and L2 peptides at higher pH values. It is possible (particularly in the case of Zn(ii)-native peptide system) that progressive deprotonation of metal-bound water molecules further destabilizes the distorted complex, but the DFT calculations do not indicate changes in energies that could account for the experimentally observed distribution.

### Secondary structure

The conformational properties of the peptides were investigated by far-UV circular dichroism (CD) spectroscopy over the pH range 3.5–11.5. Structural motifs such as α-helices, β-structures, and polyproline II (PPII) helices are commonly associated with antimicrobial activity and membrane interactions.^83^ All ligands and their Cu(ii)/Zn(ii) complexes display a characteristic band in the 202–206 nm region (Fig. S8). For the native peptide and L1, this band exhibits a negative Cotton effect, whereas for the all-d analogue L2 a positive Cotton effect is observed, consistent with its opposite chirality.^84^ The band position suggests a tendency toward PPII-type conformations typical of Pro-rich sequences.^85^

**Fig. 11:**
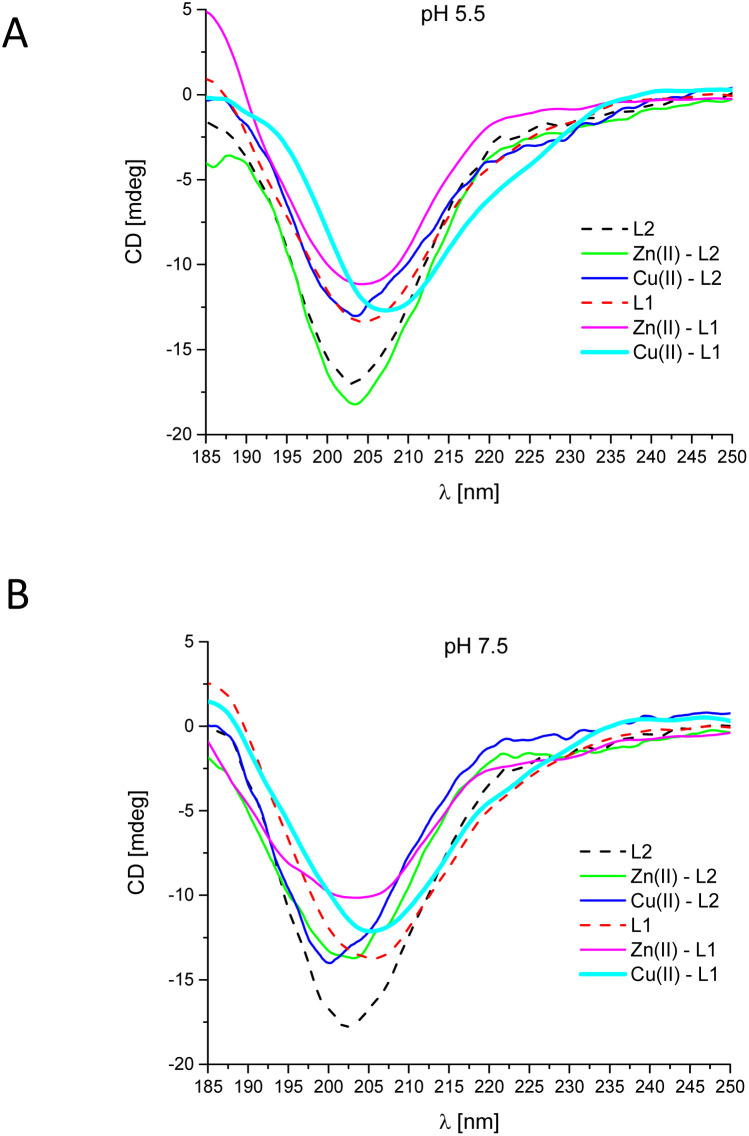
Comparison of far-UV CD spectra for the peptide ligands (A) (L1) FPNPHQPPkhPDK and (B) (L2) fpnphqppkhpdk and their Cu(ii) and Zn(ii) complexes, recorded at different pH values: (A) pH = 5.5; (B) pH = 7.5 in aqueous solution of 0.004 M HClO_4_ with ionic strength *I* = 0.1 M (NaClO_4_). [L] = 0.00035 M, molar ratio M : L = 0.9 : 1; *T* = 298 K; optical path length = 0.02 cm. The spectra of L2 and its metal complexes were multiplied by (−1) to facilitate visualization and comparison of spectral extrema between both systems.

At pH values relevant to the oral cavity (5.5 and 7.5), the spectra of the peptides and their metal complexes remain highly similar (Fig. 11 and S8), and the l- and d-based systems show the expected mirror-image relationship (Fig. S9). Overall, metal coordination does not induce major changes in the global secondary structure under these conditions. Only minor variations are observed for the Cu(ii)-L1 system. At pH 5.5, coordination results in a slight red shift of the main band and band broadening, with subtle differences around 222 nm (Fig. 11, S10A and B), suggesting a small metal-induced conformational adjustment with limited α-helical contribution. However, at pH 7.5, the spectra of free L1 and its Cu(ii) complex are nearly superimposable. For L2, Cu(ii) coordination at pH 7.5 induces only a slight shift of the Cotton-effect band in the opposite direction (Fig. 11B). In contrast, Zn(ii) complexation does not produce significant spectral changes for L1 or L2 at either pH (Fig. 11, S10C and D).

Taken together, the CD data indicate that metal binding does not substantially reorganize the peptide backbone in aqueous solution under physiologically relevant conditions, and any structural adjustments are subtle and system-dependent. To determine whether metal complexation and chirality translate into measurable differences in biological performance, the antimicrobial activity of the ligands and their Cu(ii) and Zn(ii) complexes was subsequently evaluated by MIC determination.

### Antimicrobial activity

Considering the physiological relevance of pH fluctuations in the oral cavity, which typically range from 5.3 to 7.8 depending on individual health status,^86^ the antimicrobial activity of the tested ligands and their corresponding metal complexes was systematically evaluated under two distinct pH conditions: pH 5.5 (Table 2) and pH 7.4 (Table 3). This approach allows assessment of potential pH-dependent variations in their inhibitory efficacy against microorganisms colonizing oral cavity under physiological and pathological conditions. The obtained results were collected in Tables 2 and 3.

**Table 2:** The antibacterial and anti-*Candida* activities of peptides/complexes were assessed *in vitro* by determining their MIC (µg mL^−1^). Antimicrobial tests were conducted in a 0.01 M MES buffer at pH 5.5. Experiments were performed for peptides and their copper(ii) and zinc(ii) complexes. N/D – not determined within the concentration range used in this study, >500

Strain pH 5.5	FPNPHQPPKHPDK (native^31^)			FPNPHQPPkhPDK (L1)			fpnphqppkhpdk (L2)		
	Native	+Cu(ii)	+Zn(ii)	L1	+Cu(ii)	+Zn(ii)	L2	+Cu(ii)	+Zn(ii)
*E. coli*	N/D	N/D	**500**	N/D	N/D	**500**	N/D	N/D	**500**
ATCC 25922									
*P. aeruginosa*	N/D	N/D	N/D	N/D	N/D	**500**	N/D	**500**	**500**
ATCC 15442									
*E. faecalis*	N/D	N/D	**500**	N/D	**500**	**500**	**500**	**250**	**250**
ATCC 29212									
*S. aureus*	N/D	**500**	**500**	N/D	**500**	**250**	**250**	**250**	**250**
ATCC 25923									
*S. mutans*	N/D	N/D	**500**	N/D	**250**	**250**	N/D	**250**	**125**
PCM 2502									
*S. sanguinis*	N/D	N/D	**500**	**500**	**250**	**125**	**125**	**62.5**	**62.5**
PCM 2335									
*C. albicans*	N/D	N/D	N/D	N/D	**500**	**500**	N/D	**500**	**250**
SC5314									

**Table 3:** The antibacterial and anti-*Candida* activities of peptides/complexes were assessed *in vitro* by determining their MIC (µg mL^−1^). Antimicrobial tests were conducted in a 0.01 M MES buffer at pH 7.4. Experiments were performed for peptides and their copper(ii) and zinc(ii) complexes. N/D – not determined within the concentration range used in this study, >500

Strain pH 7.5	FPNPHQPPKHPDK (native^31^)			FPNPHQPPkhPDK (L1)			fpnphqppkhpdk (L2)		
	Native	+Cu(ii)	+Zn(ii)	L1	+Cu(ii)	+Zn(ii)	L2	+Cu(ii)	+Zn(ii)
*E. coli*	N/D	N/D	N/D	N/D	N/D	N/D	N/D	N/D	N/D
ATCC 25922									
*P. aeruginosa*	N/D	N/D	N/D	N/D	N/D	N/D	N/D	N/D	N/D
ATCC 15442									
*E. faecalis*	N/D	N/D	N/D	N/D	N/D	N/D	N/D	**500**	**500**
ATCC 29212									
*S. aureus*	N/D	N/D	N/D	N/D	N/D	N/D	N/D	N/D	**500**
ATCC 25923									
*S. mutans*	N/D	N/D	N/D	N/D	**500**	**250**	N/D	**500**	**500**
PCM 2502									
*S. sanguinis*	N/D	N/D	**500**	N/D	**500**	**500**	**250**	**250**	**125**
PCM 2335									
*C. albicans*	N/D	N/D	N/D	N/D	N/D	N/D	N/D	N/D	**500**
SC5314									

The antibacterial activity was significantly enhanced under slightly acidic conditions (pH 5.5) and upon coordination with metal(II) ions. At this pH value, the free L1 and L2 ligands themselves exhibited notable antibacterial activity. Peptidomimetic L1 displayed a MIC of 500 µg mL^−1^ against *S. sanguinis*, whereas peptidomimetic L2 showed MIC values of 500 µg mL^−1^ against *E. faecalis*, 250 µg mL^−1^ against *S. aureus*, and 125 µg mL^−1^ against *S. sanguinis* (Table 2). These findings are particularly remarkable given that the native peptide displayed no detectable antimicrobial activity against these pathogens.^31^

Upon coordination with metal ions under the same conditions, the complexes demonstrated antimicrobial activity against nearly all tested pathogens. For the Cu(ii) complexes of L1, MIC values were determined primarily against Gram-positive bacteria, with the most pronounced activity observed against *S. mutans* and *S. sanguinis* (both at 250 µg mL^−1^). The Cu(ii)-L2 system exhibited the strongest antimicrobial activity against *S. sanguinis* at both tested pH values, and additionally, it was the only Cu(ii) complex showing activity against the Gram-negative bacterium (*P. aeruginosa*) at pH 5.5.

In the case of Zn(ii) complexes, the MIC values were successfully determined for both L1 and L2 against all tested microbial strains under a slightly acidic environment. Notably, antimicrobial activity against the Gram-negative bacterium *E. coli was* observed exclusively for the Zn(ii) complexes, with an MIC value of 500 µg mL^−1^. Comparative analysis revealed that Zn(ii) complexes generally exhibited enhanced antimicrobial potency relative to their Cu(ii) counterparts across most bacterial strains, suggesting a superior efficacy of zinc coordination compounds. The most pronounced antimicrobial effect was observed for the Zn(ii) complex with the only d-amino acid-containing peptidomimetic L2. Specifically, the Zn(ii)-L2 complex demonstrated an MIC of 62.5 µg mL^−1^ against *S. sanguinis*, matching the highest level of activity recorded for the Cu(ii)-L2 system and representing the most potent antimicrobial response observed in this study.

Although commonly regarded as a commensal organism and an early colonizer of the tooth surface, *S. sanguinis* has also been implicated in serious systemic infections, including bacterial endocarditis.^87,88^ As a Gram-positive anaerobic species involved in the early stages of oral microbial colonization,^88^ its dual role as both a health-associated microorganism and a potential opportunistic pathogen makes its susceptibility to the tested compounds particularly noteworthy. The low MIC values observed for *S. sanguinis* indicate that the investigated compounds are active against this species under planktonic growth conditions, highlighting their potential usefulness against oral microorganisms that may contribute to infection when the ecological balance is disturbed.^89^

An additional important aspect of this study is the antifungal activity of the tested compounds. Under mildly acidic conditions (pH 5.5), coordination with metal ions led to the emergence of notable antifungal effects, indicating that metal binding plays a crucial role in conferring antifungal activity to the peptidomimetics. In particular, coordination of metal ions to peptidomimetics containing d-amino acids induced anticandidal activity, with the lowest MIC value (250 µg mL^−1^) observed for the Zn(ii)-L2 system. Notably, neither the native peptide^31^ nor its corresponding metal complexes exhibited antifungal activity under these conditions, underscoring the superior bioactivity of the structurally modified peptidomimetics.

The obtained results suggest that the enhanced antimicrobial activity (especially in the case of the Zn(ii)-L2 complex) may be attributed to the higher content of d-amino acids in the peptidomimetic structure compared to L1. It should also be noted that Zn(ii) and Cu(ii) ions alone did not exhibit antibacterial or antifungal activity against Gram-positive bacteria, Gram-negative bacteria, or *C. albicans* at the concentrations used in the peptide complexes (0.15–1 µg mL; Table S2, S3).

The absence of major metal-induced changes in global secondary structure suggests that the enhanced antimicrobial activity is unlikely to arise from pronounced backbone reorganization in aqueous solution. Instead, the improved potency observed upon metal complexation, particularly for the Zn(ii)-L2 system under mildly acidic conditions, may reflect metal-dependent modulation of physicochemical properties such as charge distribution and conformational flexibility.

The influence of metal coordination on antimicrobial activity has been documented for numerous AMP-metal complexes and may arise through several distinct mechanisms. In Cu(ii)-peptide systems, enhanced biological activity has frequently been associated with structural rearrangements and redox-related effects. For example, the Cu(ii) complex of the shrimp-derived peptide PvHCt exhibited higher antimicrobial activity than the free peptide, which was accompanied by increased α-helical content, and ROS generation.^90^ Similar Cu-dependent mechanisms have also been proposed for histatin-5 and for Cu(ii) complexes of the C-terminal fragment of CCL-28, both of which displayed enhanced antimicrobial activity under physiologically relevant conditions.^91,92^ In contrast, Zn(ii) is generally considered to modulate biological activity through redox-independent mechanisms. Enhanced antifungal activity observed for Zn(ii)-shepherin I and Zn(ii)-pramlintide complexes has been attributed to metal-induced conformational changes and subsequent fibril formation.^93,94^ In both systems, Zn(ii) binding promoted the formation of fibrillar assemblies associated with fungal cell damage, demonstrating that metal coordination may substantially alter biological activity through changes in peptide organization and physicochemical properties.

Besides the direct effects of metal coordination, peptide stereochemistry may also play an important role in determining biological activity. Previous studies on MUC7-derived peptidomimetics demonstrated that even minimal stereochemical modifications can significantly alter metal-binding properties, conformational behaviour, antimicrobial activity, and resistance to proteolytic degradation. In particular, selective substitution of two amino acids with their d-counterparts converted an inactive MUC7-derived peptide into a selective metal-dependent antimicrobial agent active against oral streptococci, highlighting the complex interplay between peptide stereochemistry, conformational flexibility, and metal coordination.^95^ Interestingly, studies on other MUC7-derived peptidomimetics containing d-amino acid and retro-inverso modifications demonstrated that stereochemical editing can substantially alter Zn(ii)-binding behaviour, and antimicrobial activity,^34^ although the direction and magnitude of these effects appear to be strongly sequence-dependent.

Within this context, the behaviour of the Zn(ii)-L2 system appears particularly noteworthy. Since all investigated ligands possess identical amino-acid compositions and net charges, the superior activity of Zn(ii)-L2 cannot be readily attributed to differences in overall charge. Instead, it most likely arises from the combined effect of complete d-amino acid substitution and Zn(ii) coordination. While the all-d analogue already exhibits enhanced antimicrobial activity and metabolic stability, Zn(ii) binding may further modulate its local geometry, conformational flexibility, and interactions with microbial targets, resulting in an approximately two-fold increase in activity. Thus, although Zn(ii) coordination does not trigger a major structural rearrangement, it appears to provide a biologically relevant enhancement of the antimicrobial properties of the L2 peptidomimetic.

To further evaluate the therapeutic potential of the most active systems, their cytotoxicity and hemolytic activity were subsequently assessed.

### Cytotoxicity

Cytotoxicity toward NHDF cells was assessed using the MTT assay, and the results are summarized in Table 4 All tested peptides and their Cu(ii) and Zn(ii) complexes demonstrated low cytotoxicity after 24 h incubation at 500 µg mL^−1^, with cell viability values ranging from 92.1% to 96.4%. The native peptide FPNPHQPPKHPDK showed high viability (96.4%), while its Cu(ii) and Zn(ii) complexes maintained similarly favorable profiles (94.2% and 95.5%, respectively). Analogously, L1 and L2, as well as their corresponding metal complexes, exhibited comparably high viability values (92.1–95.4%), indicating that neither sequence modification nor metal coordination resulted in increased toxicity toward NHDFs.

**Table 4:** Cell viability assay. NHDF cells were plated in a 96-well titer plate with 500 µg mL^−1^ of peptides and Cu(ii) and Zn(ii) complexes and incubated for 24 h. Percentage of cell viability was determined by MTT assay. The results represent the averages of triplicate experiments ± SD

Compound	Cell viability (%)
FPNPHQPPKHPDK (native)	96.4 ± 1.2
Cu(ii)-native	94.2 ± 0.8
Zn(ii)-native	95.5 ± 1.2
FPNPHQPPkhPDK (L1)	95.3 ± 1.6
Cu(ii)-L1	93.2 ± 1.1
Zn(ii)-L1	95.4 ± 0.2
fpnphqppkhpdk (L2)	94.5 ± 1.3
Cu(ii)-L2	92.1 ± 0.6
Zn(ii)-L2	94.6 ± 0.8

Overall, the consistently high viability observed for all tested systems suggests good compatibility with mammalian cells at the tested concentration. Such behavior is in agreement with previous studies reporting that short antimicrobial peptide analogues and their metal complexes can retain biological activity while exhibiting limited cytotoxic effects toward normal cells.^96,97^

### Hemolytic activity

Hemolytic activity of the investigated peptides and their Cu(ii) and Zn(ii) complexes was evaluated using sheep red blood cells, and the results are presented in Table 5. All tested compounds exhibited very low hemolytic activity at the examined concentration, with hemolysis values ranging from 1% to 3%. The native peptide FPNPHQPPKHPDK induced only minimal hemolysis (1.3 ± 0.2%), and similarly low values were observed for its Cu(ii) and Zn(ii) complexes (2.2 ± 0.4% and 1.4 ± 0.1%, respectively). The analogues L1 and L2, together with their corresponding metal complexes, also showed negligible erythrocyte disruption, with hemolysis remaining at or below 3.4%.

**Table 5:** Hemolytic activity of peptides and their Cu(ii) and Zn(ii) complexes. Values are mean ± SD of three independent experiments

Compound	Hemolysis (%)
FPNPHQPPKHPDK (native)	1.3 ± 0.2
Cu(ii)-native	2.2 ± 0.4
Zn(ii)-native	1.4 ± 0.1
FPNPHQPPkhPDK (L1)	3.1 ± 0.1
Cu(ii)-L1	3.1 ± 0.2
Zn(ii)-L1	2.4 ± 0.3
fpnphqppkhpdk (L2)	3.2 ± 0.1
Cu(ii)-L2	3.4 ± 0.4
Zn(ii)-L2	2.6 ± 0.1

These results indicate that neither peptide modification nor metal coordination significantly affected membrane compatibility toward erythrocytes. In combination with the high NHDF cell viability observed in the cytotoxicity assay, the data suggest that the investigated peptidomimetic systems possess a favorable safety profile toward mammalian cells at the tested concentration, supporting their further evaluation as antimicrobial candidates.

### Proteolytic stability

Peptide-based antimicrobials are inherently susceptible to proteolytic degradation by enzymes present in biological fluids, including human saliva and blood. In the oral cavity, proteases derived from salivary glands, gingival crevicular fluid, host tissues, and oral microbiota may rapidly compromise peptide stability and biological activity.^98,99^ Similarly, endogenous proteases in plasma and serum significantly limit peptide persistence and bioavailability.^100^ Therefore, improving proteolytic resistance is essential for the practical application of peptide-based agents. To evaluate the intrinsic stability of the investigated sequences, the free peptides were subjected to trypsin digestion and incubated in human plasma under physiologically relevant conditions.

Two mucin-derived peptidomimetics, FPNPHQPPkhPDK and fpnphqppkhpdk, were evaluated for their susceptibility to proteolytic degradation under biologically relevant conditions. Trypsin was used as a model of trypsin-like proteases present in the oral cavity, while additional experiments assessed peptide stability in human plasma to reflect systemic proteolytic environments.

As trypsin selectively cleaves peptide bonds at the C-terminal side of lysine and arginine residues,^101,102^ both present in the investigated sequences, digestion experiments were performed to compare the stability of the native peptide and its peptidomimetic analogues. The native peptide, composed exclusively of l-amino acids, was readily cleaved by trypsin, consistent with our previous report^31^ and confirmed here by multiple signals in the MALDI-TOF MS spectrum. A characteristic fragment at *m*/*z* 496.253, corresponding to HPDK (Fig. S11A), indicates enzymatic cleavage at the most susceptible site. In contrast, this signal was absent in the spectra of the peptidomimetics containing d-amino acids (L1 and L2, Fig. S11B and C, respectively), demonstrating that substitution with d-amino acids effectively enhances resistance to enzymatic degradation at this site.

**Fig. 12:**
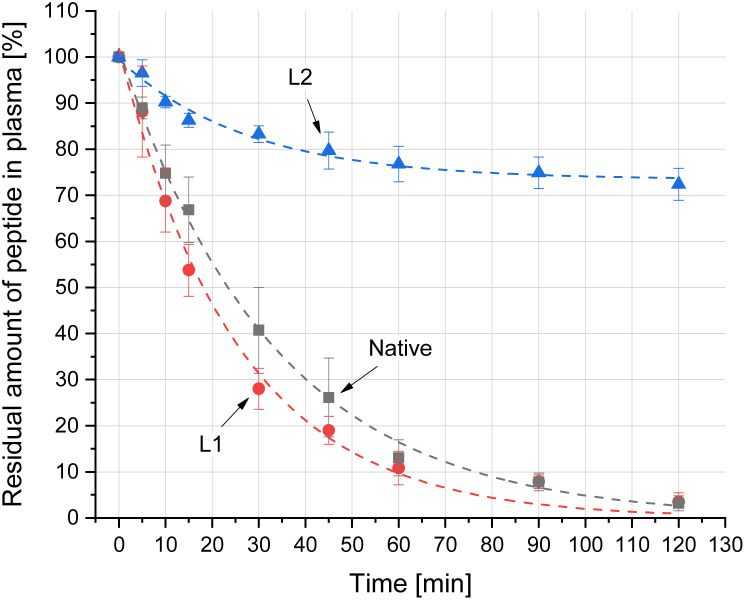
Comparison of the enzymatic stability of peptides: (L1) FPNPHQPPkhPDK (red), (L2) fpnphqppkhpdk (blue), and the native peptide FPNPHQPPKHPDK (black). [L] = 0.0001 M. Reactions were terminated by the addition of 0.5 M HClO_4_. 8 mM phenylalaninol was used as an internal standard. Incubation was performed at 37 °C. with 0.1% trifluoroacetic acid. Plasma samples used for all measurements shared the same identifier to ensure consistency. Each sample was analyzed in four independent replicates.

To further assess stability under physiologically relevant conditions, peptide degradation was evaluated in human plasma at 37 °C over a 120 min incubation period. Residual peptide integrity was monitored at multiple time points to determine degradation kinetics (Fig. 12 and S12).

The native l-amino-acid peptide was rapidly degraded, with a calculated half-life of 23 ± 5 min, and only 6% of the initial peptide remaining after 120 min (Table 6). The peptidomimetic L1, containing two d-amino-acid residues, exhibited a comparable degradation profile, with an estimated half-life of 16 ± 2 min. These findings indicate that, unlike in the case of trypsin digestion, partial d-amino-acid substitution does not confer enhanced resistance in plasma. This behavior likely reflects the action of multiple *endo*- and *exo*-peptidases present in plasma, which cleave alternative susceptible l-amino-acid sites.^103,104^

**Table 6:** A summary of the residual enzymatic activity of the studied peptides over time. The experiments were conducted using human plasma samples. Measurements were performed using a UV detector at *λ* = 200 nm. The peptide, at a concentration of [L] = 0.1 mM, was dissolved in 0.01 M ammonium acetate buffer at pH 7.4. Phenylalaninol at a concentration of 8 mM served as an internal standard. Mobile phase consisted of acetonitrile and water (13 : 87, v/v), supplemented with 0.1% trifluoroacetic acid. Enzymatic reactions were terminated with 0.5 M HClO_4_. Peptide solutions were maintained at 37 °C using a water bath. Plasma samples used for all measurements shared the same identifier to ensure consistency. Each sample was analyzed in four independent replicates. *t*_1/2_ – corresponds to half residual enzymatic activity

Ligand	Residual enzymatic activity in human plasma [%]									
	0 min	5 min	10 min	15 min	30 min	45 min	60 min	90 min	120 min	*t*_1/2_
FPNPHQPPKHPDK (native)	100	89 ± 2	75 ± 6	67 ± 7	41 ± 9	26 ± 8	13 ± 4	7 ± 2	3 ± 2	23 ± 5
FPNPHQPPkhPDK (L1)	100	88 ± 9	69 ± 7	54 ± 6	28 ± 5	19 ± 3	11 ± 4	8 ± 2	4 ± 2	16 ± 2
fpnphqppkhpdk (L2)	100	96 ± 3	90 ± 1	86 ± 2	83 ± 2	80 ± 4	77 ± 4	75 ± 3	72 ± 3	—

In contrast, peptide L2 demonstrated markedly enhanced resistance to proteolytic degradation (Fig. 12, S12C). Over the 120 min incubation period, only a ∼25% decrease in peptide content was observed, and no reliable half-life could be determined due to the minimal extent of degradation. The degradation profile approached a plateau after prolonged incubation. The improved stability of L2 can be attributed to its all-d-amino-acid composition, as proteases capable of efficiently hydrolyzing peptide bonds involving d-residues are rare in multicellular organisms.^105^ Additionally, incorporation of d-amino acids may induce altered backbone conformations that reduce enzymatic recognition and cleavage susceptibility.^106–108^

Collectively, these findings indicate that while partial d-amino-acid substitution does not confer plasma stability, full d-residue incorporation markedly enhances resistance to proteolytic degradation under systemic conditions.

## Conclusions

In this study, we systematically explored the coordination chemistry, structural behavior, proteolytic stability, and biological activity of mucin-derived peptidomimetics containing d-amino acids and their Cu(ii) and Zn(ii) complexes. Potentiometric, spectroscopic, and DFT analyses demonstrated that metal binding occurs through donor sets analogous to those of the native peptide, inducing only subtle local conformational adjustments without major global structural reorganization. Circular dichroism measurements further confirmed that secondary-structure variations upon complexation are minor, with only limited α-helical contribution observed for the Cu(ii)-L1 system.

Despite these modest structural effects, d-amino-acid incorporation exerts a profound influence on biological performance. Peptidomimetics containing d-residues exhibit markedly enhanced antimicrobial activity compared to the native peptide, with the fully d-configured analogue (L2) showing the highest potency. This enhancement is particularly pronounced under mildly acidic conditions (pH 5.5), relevant to the oral environment, where coordination of Zn(ii) and Cu(ii) further amplifies antibacterial efficacy and induces antifungal activity not observed for the native system.

Proteolytic stability studies reveal a clear chirality-dependent trend: whereas the native peptide and the partially modified L1 analogue display comparable plasma half-lives (23 and 16 min, respectively), the fully d-amino-acid peptidomimetic L2 exhibits substantially improved resistance, with only ∼25% degradation after 2 h. These findings demonstrate that full enantiomeric substitution is required to achieve meaningful protection against systemic proteolysis. Importantly, all investigated peptidomimetics and their metal complexes exhibit minimal hemolytic activity and low cytotoxicity toward NHDF cells, confirming a favorable safety profile.

Notably, the strategically modified peptidomimetics outperform the naturally generated HPDK fragment in terms of antimicrobial efficacy, indicating that selective d-amino acid substitution can provide greater functional benefits than native proteolytic processing.

Collectively, our results highlight the complementary roles of d-amino-acid incorporation and metal coordination in enhancing both stability and antimicrobial potency. This combined strategy offers a promising framework for the development of metal-assisted mucin-derived peptide therapeutics targeting oral pathogens and associated systemic infections.

## Author contributions

Anna Ślusarczyk: formal analysis, data curation, writing – original draft, investigation, editing. Denise Bellotti: supervision, methodology, software, data curation, validation. Silvia Leveraro: supervision, investigation, visualization. Tomasz Janek: formal analysis, data curation, writing – original draft, investigation. Fabio Zobi: methodology, software, writing – original draft, validation. Maurizio Remelli: validation, review & editing. Joanna Wątły: conceptualization, funding acquisition, formal analysis, methodology, writing – original draft, writing – review and editing, supervision, investigation and validation.

## Conflicts of interest

There are no conflicts to declare. The manuscript was written through contributions of all authors. All authors have given approval to the final version of the manuscript. These authors contributed equally.

## Supplementary Material

RA-016-D6RA02768G-s001

## Data Availability

The data supporting this study are available within the article and its supplementary information (SI). Biological activity data are also included in the main text and SI. All raw datasets will be deposited in the institutional repository RODBUK (Cracow Open Research Data Repository of the University of Wrocław) and will be made publicly available upon publication. A persistent DOI will be provided. Supplementary information: certificates of analysis of synthesized peptides, potentiometric titrations, competition plots, mass spectrometry data, far-UV CD and EPR spectra, DFT optimized structures, enzymatic stability (HPLC) data, and trypsin digestion assays. See DOI: https://doi.org/10.1039/d6ra02768g.
