# Supplementary material for: Beyond proteolysis: rational modification of mucin-derived peptidomimetics with enhanced metal-mediated antimicrobial activity
Source: RSC Adv. 2026 Jul 9. Online ahead of print. doi: 10.1039/d6ra02768g (PMC13347497; doi:10.1039/d6ra02768g)
Supplement: RA-OLF-D6RA02768G-s001 [file RA-OLF-D6RA02768G-s001.pdf]

Supplementary Information (SI) for RSC Advances.
This journal is © The Royal Society of Chemistry 2026

Supporting Information for:

Beyond Proteolysis: Rational Modification of Mucin-Derived Peptidomimetics with Enhanced Metal-Mediated Antimicrobial Activity

Anna Ślusarczyk [a], Denise Bellotti [b], Silvia Leveraro [b], Tomasz Janek [c], Fabio Zobi [d], Maurizio Remelli [b], Joanna Wątły [a,*]

[a] Faculty of Chemistry, University of Wrocław, F. Joliot-Curie 14, 50-383 Wrocław, Poland. anna.slusarczyk@uwr.edu.pl; *joanna.watly2@uwr.edu.pl
[b] Department of Chemical, Pharmaceutical and Agricultural Sciences, University of Ferrara, Via Luigi Borsari 46, 44121, Ferrara, Italy. denise.bellotti@unife.it; silvia.leveraro@unife.it; maurizio.remelli@unife.it
[c] Department of Biotechnology and Food Microbiology, Wrocław University of Environmental and Life Sciences, Chełmońskiego 37, 51-630, Wrocław, Poland. tomasz.janek@upwr.edu.pl
[d] Department of Chemistry, Fribourg University, Chemin Du Musée 9, 1700 Fribourg, Switzerland. fabio.zobi@unifr.ch

***corresponding author**

Table of contents:

Figure S 1. Certifications of analysis of synthesized peptides (A) L1 (FPNPHQPPkhPDK) and B) L2 (fpnphqppkhpdk) received from KareBayTM Biochem company. 4

Figure S 2 . Species distribution for the ligands: (A) L1 (FPNPHQPPkhPDK) and B) L2 (fpnphqppkhpdk) as a function of pH in aqueous solution containing 0.004 mM $HClO_4$, ionic strength I = 0.1 M $NaClO_4$, T = 25 °C, [L] = 0.0004 M. 4

Table S 1. m/z values obtained from the ESI-MS experiment for the L1 peptide, its complexes with Cu(II) and Zn(II) ions, and the adducts with sodium and potassium ions. 4

Figure S 3. MS spectra of complexes with ligand 1 (FPNPHQPPkhPDK): Cu(II) – L1 system (A) and : Zn(II) – L1 system (B). Comparison of experimental and simulated spectra are presented for selected signals. Molar ratio M:L – 1:1. [L] = 0.0001 M. Samples prepared in MeOH:H_2O (50:50) mixture. 6

Figure S 4. MS spectra of complexes with ligand 2 (fpnphqppkhpdk): Cu(II) – L2 system (A) and B: Zn(II) – L2 system (B). Comparison of experimental and simulated spectra are presented for selected signals. Molar ratio M:L – 1:1. [L] = 0.0001 M. Samples prepared in MeOH:H_2O (50:50) mixture. 7

Figure S 5. The EPR spectra of the (A) Cu(II) – L1(FPNPHQPPkhPDK) and (B) Cu(II) – L2 (fpnphqppkhpdk) complexes recorded in an aqueous solution containing 0.004 M $HClO_4$ and 0.1 M $NaClO_4$ (ionic strength), with the addition of 25% ethylene glycol as a cryoprotectant. T = 77.36 K; X-band region (approximately 9.6 GHz). [Cu(II)] = 0.001 M, the molar ratio of metal to ligand 0.9:1. 8

Figure S 6. Competition plot between L1 (FPNPHQPPkhPDK), L2 (fpnphqppkhpdk), and native peptide (FPNPHQPPKHPDK)[1] with Cu(II) ions describing complex formation at different pH values in a hypothetical situation, in which equimolar amounts of all the reagents are mixed. Conditions: T = 25 °C, [Cu(II)] = [L1] = [L2] = [native] = 0.001 M. 8

Figure S 7. DFT calculated structures for Cu(II) – native (FPNPHQPPKHPDK) complex species: (A) $[CuH_2L]^{2+}$and (B) [CuL]. Arrows indicate intramolecular H-bonds. 9

Figure S 8. Far-UV CD spectra for (A) native (FPNPHQPPKHPDK), (B) L1 (FPNPHQPPkhPDK), (C) L2 (fpnphqppkhpdk) ligands; (D) Cu(II)-native, (E)Cu(II)- L1,(F) Cu(II)-L2; (G) Zn(II) – native, (H) Zn(II) – L1, and (I) Zn(II) – L2 complexes in aqueous solution of 0.004 M $HClO_4$ with ionic strength I = 0.1 M ($NaClO_4$). [L] = 0.00035 M, molar ratio M:L = 0.9:1; T = 298 K; optical path length = 0.02 cm. 10

Figure S 9. Comparison of far-UV CD spectra for the peptide ligands: native (FPNPHQPPKHPDK), L1 (FPNPHQPPkhPDK) and L2 (fpnphqppkhpdk) and their Cu(II) and Zn(II) complexes, recorded at pH values: (A) 5.5 and (B) 7.5 in aqueous solution of 0.004 M $HClO_4$ with ionic strength I = 0.1 M ($NaClO_4$). [L] = 0.00035 M, molar ratio M:L = 0.9:1; T = 298 K; optical path length = 0.02 cm. 10

Figure S 10. Comparison of far-UV CD spectra for the peptide complexes: (A) Cu(II) - L1 and Cu(II) - L2 at pH 5.5, (B) Cu(II) - L1 and Cu(II) - L2 at pH 7.5, (C) Zn(II) - L1 and Zn(II) - L2 at pH 5.5 and (D) Zn(II) - L1 and Zn(II) - L2 at pH 7.5 in aqueous solution of 0.004 M $HClO_4$ with ionic strength I = 0.1 M ($NaClO_4$). [L] = 0.00035 M, molar ratio M:L = 0.9:1; T = 298 K; optical path length = 0.02 cm. 10

Figure S 11. MALDI-TOF MS spectra of (A) FPNPHQPPKHPDK (native peptide), (B) FPNPHQPPkhPDK (L2, 2 D-aa), and (C) fpnphqppkhpdk (L3, all D-aa) peptides after trypsin digestion. Each sample consists of peptide, phosphate buffer, and trypsin. The signal at m/z 496.253 corresponds to the HPDK fragment; this signal is absent in the peptidomimetics (B and C), indicating proteolytic resistance at the modified site in the peptide chain. 12

Figure S 12. Relative activity of the (A) FPNPHQPPKHPDK (native peptide), (B) FPNPHQPPkhPDK (L2, 2 D-aa), and (C) fpnphqppkhpdk (L3, all D-aa) fpnphqphkpdk peptide in plasma over time. Data are presented as mean values with error bars indicating variability between measurements. peptide in plasma over time. UV detection at λ=200 nm, [L] = 0.1 mM in aqueous solution of 0.01 M ammonium acetate buffer (pH 7.4); phenylalaninol (8 mM) was used as an internal standard, acetonitrile/water (13:87, v/v) with 0.1% TFA as the mobile phase, and reactions were quenched with 0.5 M $HClO_4$. Samples were incubated at 37 °C and analyzed using plasma from the same donor. 13

A

KareBay Biochem, Inc.

CERTIFICATION OF ANALYSIS

Product Information:

Product Name	
Cat. NO.	825838
Lot:	23040613
Size:	100mg*1
Sequence:	FPN PHQPP KHPDK (K and H D-amino acids)
Molecular Weight:	1538.71
Storage:	-20℃

Analysis Summary:

Test Items	Standard	Result
HPLC Trace:	≧95.00%	95.68%
Mass Spectrometry:	Consistent	Consistent
Appearance:	White powder	White powder

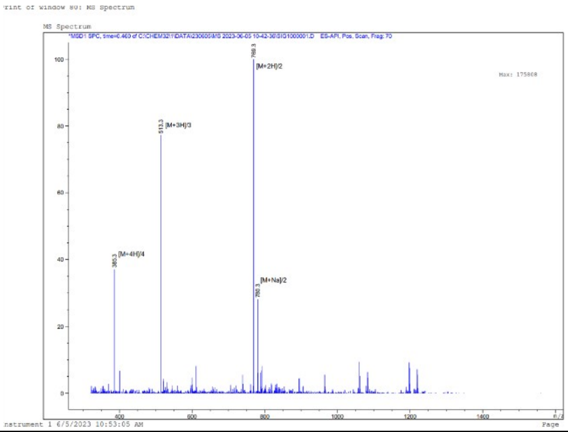

KareBay Biochem, Inc.

825838 HPLC Analysis Report

Sequence: FPN PHQPP KHPDK (K and H D-amino acids)
Sample ID:825838
Lot:23040613
Buffer A:0.1% TFA in 100% water(v/v)
Buffer B:0.1% TFA in 100% acetonitrile(v/v)
Gradient:10-80% Buffer B in 20min
Flow:1ml/min Wavelength:220nm
Column: Agilent Pursuit 5um C18 4.6*250mm

Chromatogram

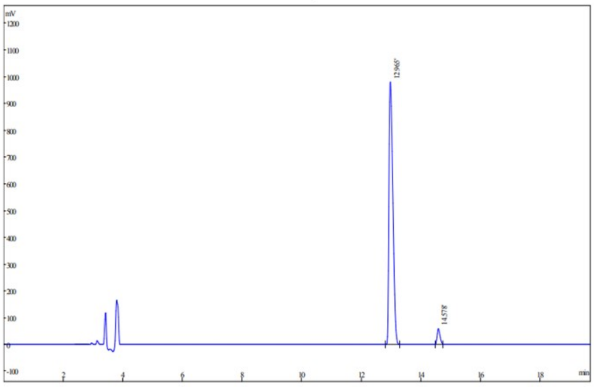

Rank	Time	Quantity	Area	Height
1	12.965	95.68	9047056	985780
2	14.578	4.324	408938	60104
Total		100	9455994	1045884

B

KareBay Biochem, Inc.

CERTIFICATION OF ANALYSIS

Product Information:

Product Name	
Cat. NO.	825837
Lot:	23040612
Size:	100mg*1
Sequence:	FPN PHQPP KHPDK (all D-amino acids)
Molecular Weight:	1538.71
Storage:	-20℃

Analysis Summary:

Test Items	Standard	Result
HPLC Trace:	≧95.00%	95.80%
Mass Spectrometry:	Consistent	Consistent
Appearance:	White powder	White powder

KareBay Biochem, Inc.

825837 HPLC Analysis Report

Sequence: FPN PHQPP KHPDK (all D-amino acids)
Sample ID:825837
Lot:23040612
Buffer A:0.1% TFA in 100% water(v/v)
Buffer B:0.1% TFA in 100% acetonitrile(v/v)
Gradient:10-80% Buffer B in 20min
Flow:1ml/min Wavelength:220nm
Column: Agilent Pursuit 5um C18 4.6*250mm

Chromatogram

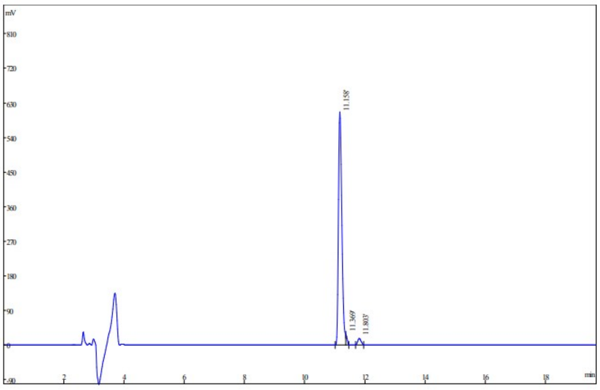

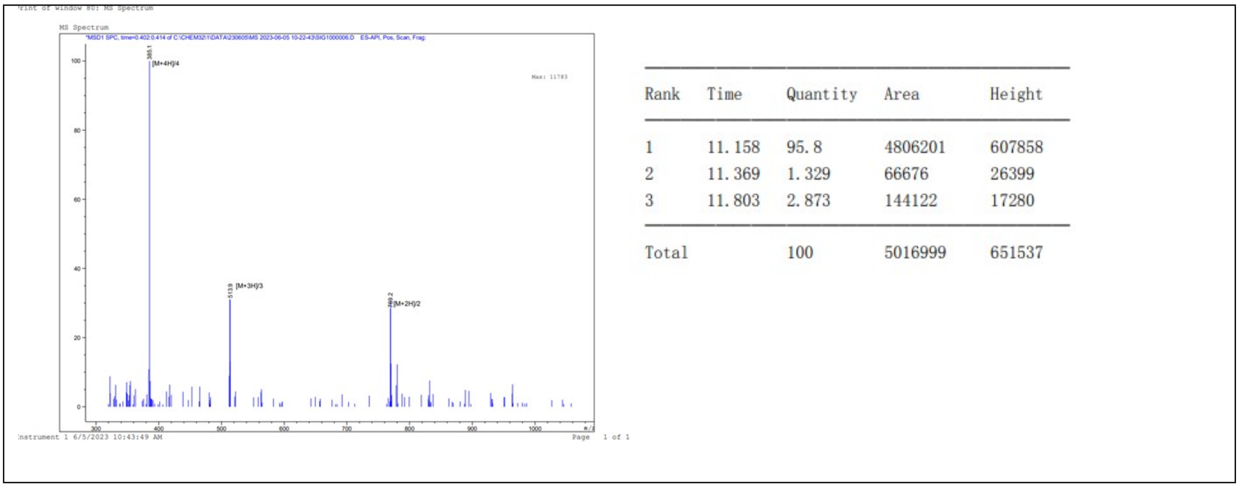

Figure S 1. Certifications of analysis of synthesized peptides (A) L1 (FPNPHQPPkhPDK) and B) L2 (fpnphqppkhpdk) received from KareBayTM Biochem company.

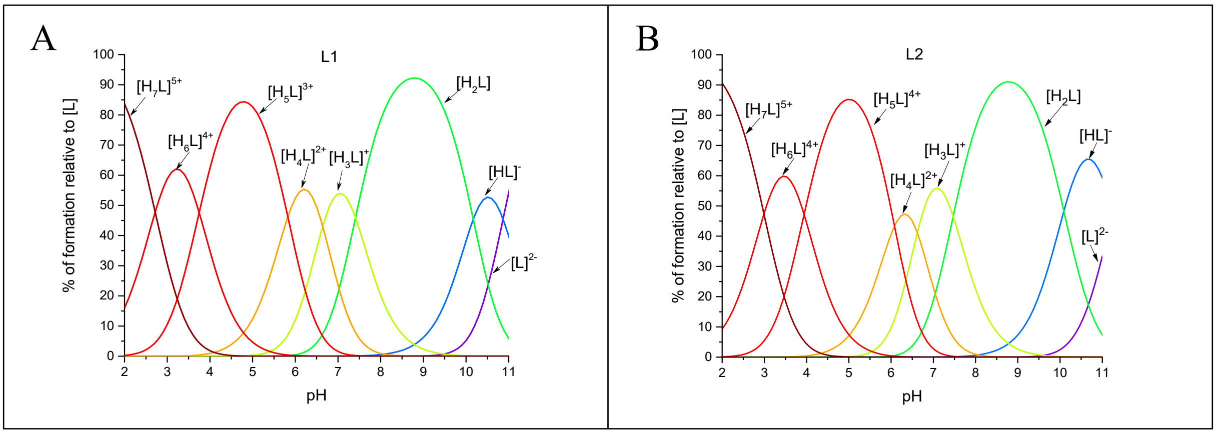

Figure S 2 . Species distribution for the ligands: (A) L1 (FPNPHQPPkhPDK) and B) L2 (fpnphqppkhpdk) as a function of pH in aqueous solution containing 0.004 mM $HClO_4$, ionic strength I = 0.1 M $NaClO_4$, T = 25 °C, [L] = 0.0004 M.

Table S 1. m/z values obtained from the ESI-MS experiment for the L1 peptide, its complexes with Cu(II) and Zn(II) ions, and the adducts with sodium and potassium ions.

form	*m/z* value
L1 (FPNPHQPPkhPDK)	
$[L]^{2+}$	770.362
$[L+Na^+]^{2+}$	781.354
$[L+K^+]^{2+}$	789.407
$[L+ Na^++2K^+]^{2+}$	820.452
$[L]^{3+}$	513.911
$[L+Na^+]^{3+}$	521.238
$[L+K^+]^{3+}$	526.607
$[L+Na^++2K^+]^{3+}$	547.304
$[L]^{4+}$	385.685
$[L+K^+]^{4+}$	395.208
Cu(II) – L1	
$[CuL]^{2+}$	801.127
$[CuL+Na^+]^{2+}$	812.118
$[CuL]^{3+}$	534.421
$[CuL+Na^+]^{3+}$	541.748
$[CuL+K^+]^{3+}$	547.117
$[CuL]^{4+}$	401.067

Zn(II) – L1	
$[ZnL]^{2+}$	802.059
$[ZnL]^{3+}$	535.042
$[ZnL]^{4+}$	401.533
L2 (fpnphqppkhpdk)	
$[L]^{2+}$	770.401
$[L+ClO_4^-+2K^++Na^+]^{2+}$	819.872
$[L]^{3+}$	513.600
$[L+Na^+]^{3+}$	521.238
$[L+ClO_4^-]^{3+}$	547.397
$[L]^{4+}$	385.701
Cu(II) – L2	
$[CuL]^{2+}$	800.862
$[CuL+K^+]^{2+}$	820.371
$[CuL+ClO_4^-]^{2+}$	850.333
$[CuL+ClO_4^-+K^+]^{2+}$	870.352
$[CuL]^{3+}$	534.210
$[CuL+ClO_4^-]^{3+}$	567.332
$[CuL+ClO_4^-+K^++Na^+]^{3+}$	588.192
$[CuL]^{4+}$	400.933
$[CuL+Na^+]^{4+}$	406.562
Zn(II) – L2	
$[ZnL]^{2+}$	802.043
$[ZnL]^{3+}$	534.232
$[ZnL+ClO_4^-]^{3+}$	568.517
$[ZnL]^{4+}$	401.525

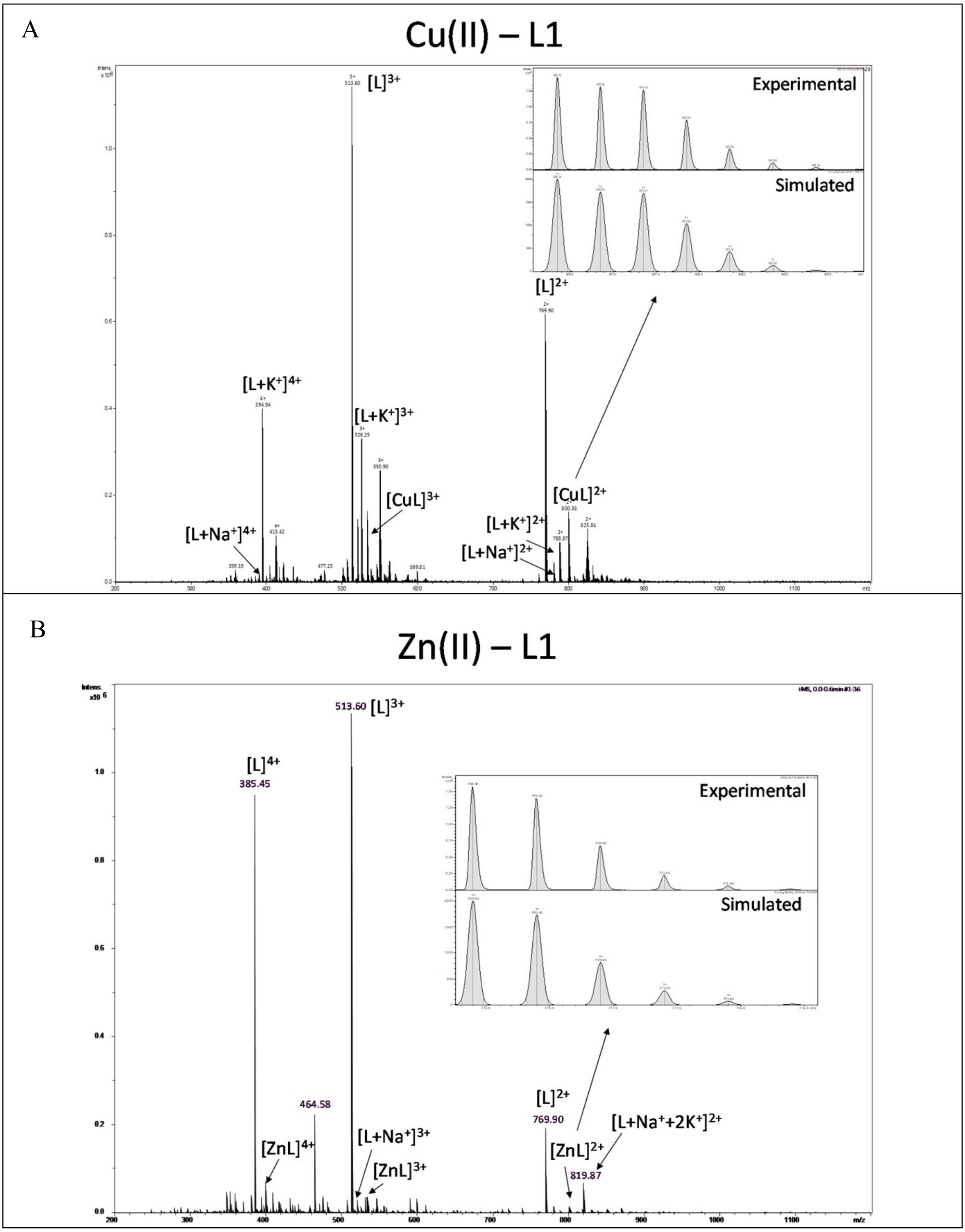

Figure S 3. MS spectra of complexes with ligand 1 (FPNPHQPPkhPDK): Cu(II) – L1 system (A) and : Zn(II) – L1 system (B). Comparison of experimental and simulated spectra are presented for selected signals. Molar ratio M:L – 1:1. [L] = 0.0001 M. Samples prepared in MeOH:H_2O (50:50) mixture.

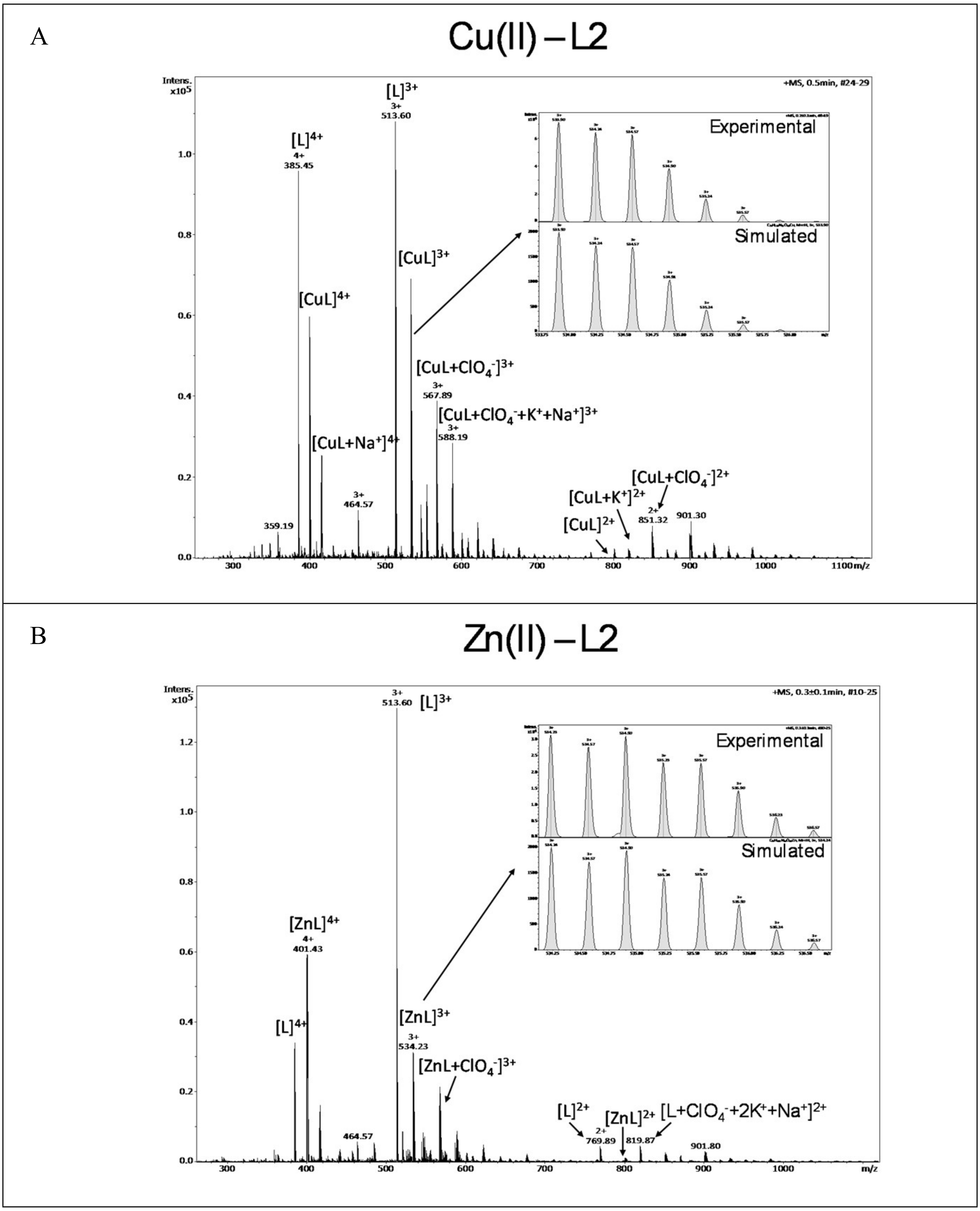

Figure S 4. MS spectra of complexes with ligand 2 (fpnphqppkhpdk): Cu(II) – L2 system (A) and B: Zn(II) – L2 system (B). Comparison of experimental and simulated spectra are presented for selected signals. Molar ratio M:L – 1:1. [L] = 0.0001 M. Samples prepared in MeOH:H_2O (50:50) mixture.

pH	A‖[G] (A_{zz})	g‖ (g_{zz})	MW Frequency [GHz]	Suggested coordination mode
3.0	121.3	2.42	9.6014	4O
4.0	123.4<br>155.7	2.42<br>2.33	9.6002	4O<br>1N
5.0	151.7	2.29	9.6012	1N
6.0	161.8	2.28	9.6000	2N

A

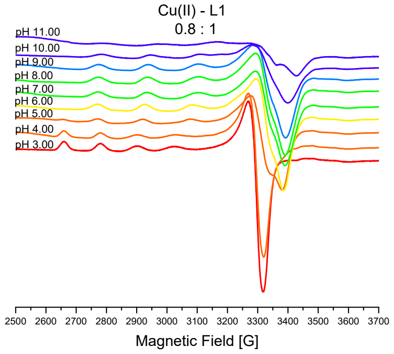

7.0	165.1	2.27	9.5993	2N
8.0	167.9	2.27	9.6007	2N
9.0	163.9	2.27	9.5981	2N
10.0	163.8<br>171.9	2.26<br>2.23	9.6011	2N<br>3N
11.0	182.9	2.23	9.6000	3N

B

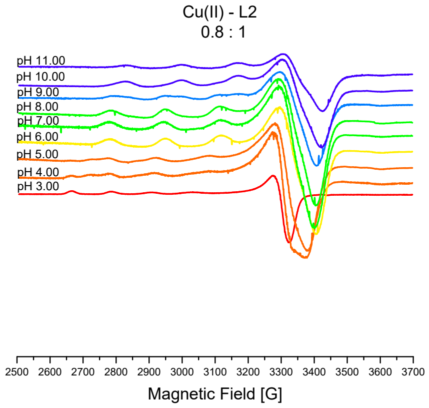

pH	A‖ [G] (A_{zz})	g‖ (g_{zz})	MW Frequency [GHz]	Suggested coordination mode
3.0	126.3	2.31	9.623962	1N
4.0	164.6<br>132.5	2.28<br>2.27	9.614770	2N<br>2N
5.0	164.0	2.27	9.616081	2N
6.0	167.3	2.26	9.615740	2N
7.0	167.3	2.26	9.616983	2N
8.0	168.2	2.26	9.618131	2N
9.0	164.2<br>170.2	2.23<br>2.24	9.613849	3N<br>3N
10.0	172.9	2.23	9.618068	3N
11.0	172.8	2.23	9.617406	3N

Figure S 5. The EPR spectra of the (A) Cu(II) – L1(FPNPHQPPKkhPDK) and (B) Cu(II) – L2 (fpnphqppkhpdk) complexes recorded in an aqueous solution containing 0.004 M $HClO_4$ and 0.1 M $NaClO_4$ (ionic strength), with the addition of 25% ethylene glycol as a cryoprotectant. T = 77.36 K; X-band region (approximately 9.6 GHz). [Cu(II)] = 0.001 M, the molar ratio of metal to ligand 0.9:1.

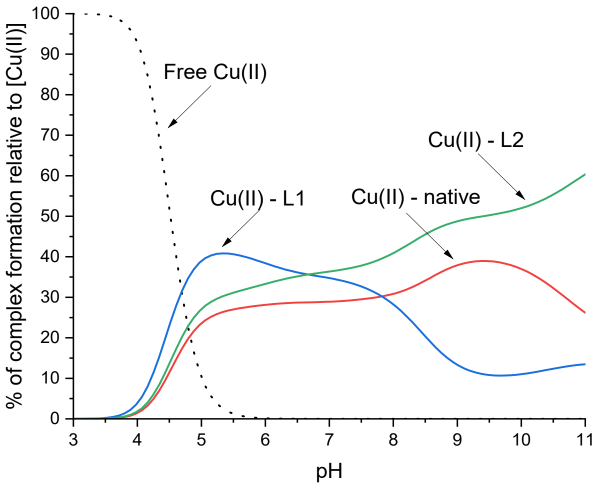

Figure S 6. Competition plot between L1 (FPNPHQPPKkhPDK), L2 (fpnphqppkhpdk), and native peptide (FPNPHQPPKKHPDK)[1] with Cu(II) ions describing complex formation at different pH values in a hypothetical situation, in which equimolar amounts of all the reagents are mixed. Conditions: T = 25 °C, [Cu(II)] = [L1] = [L2] = [native] = 0.001 M.

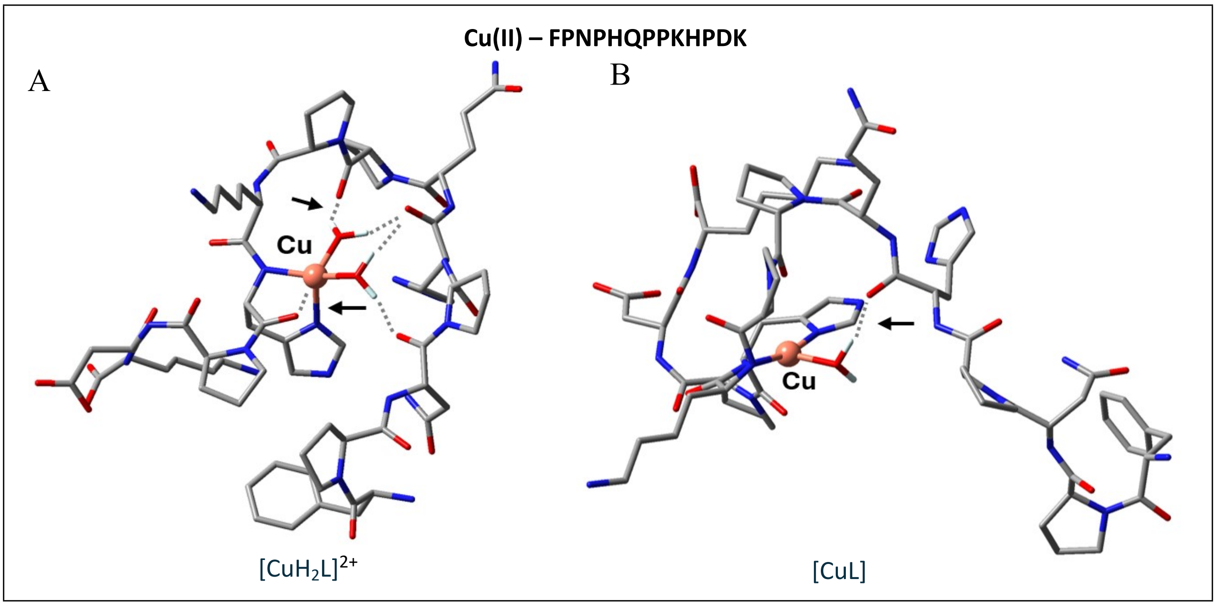

Figure S 7. DFT calculated structures for Cu(II) – native (FPNPHQPPKHPDK) complex species: (A) $[CuH_2L]^{2+}$ and (B) [CuL]. Arrows indicate intramolecular H-bonds.

Ligands

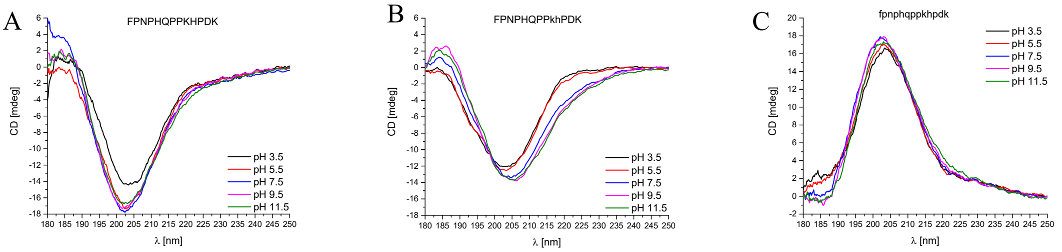

Cu(II) complexes

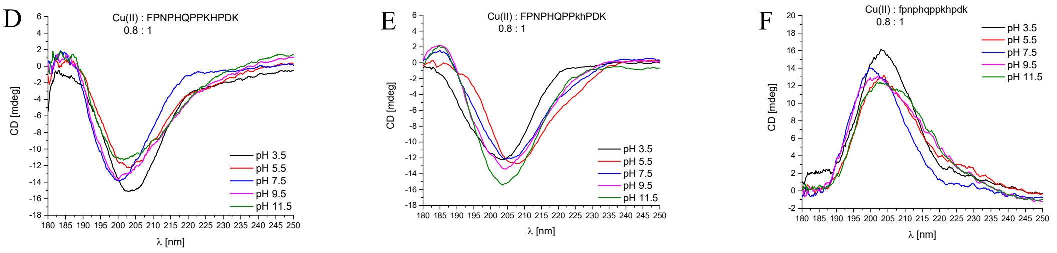

Zn(II) complexes

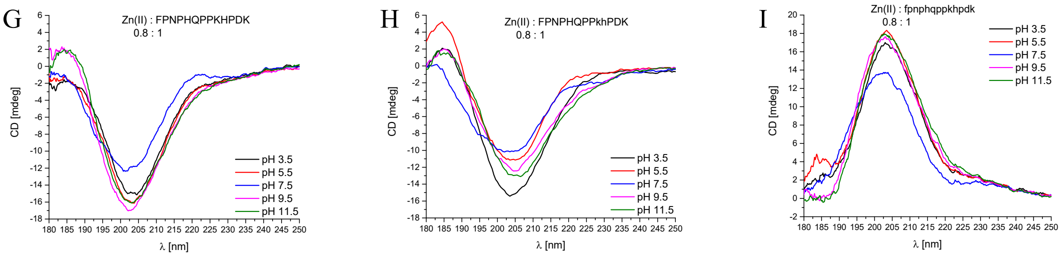

Figure S 8. Far-UV CD spectra for (A) native (FPNPHQPPKHPDK), (B) L1 (FPNPHQPPkhPDK), (C) L2 (fpnphqppkhpdk) ligands; (D) Cu(II)-native, (E)Cu(II)- L1,(F) Cu(II)-L2; (G) Zn(II) – native, (H) Zn(II) – L1, and (I) Zn(II) – L2 complexes in aqueous solution of 0.004 M $HClO_4$ with ionic strength I = 0.1 M ($NaClO_4$). [L] = 0.00035 M, molar ratio M:L = 0.9:1; T = 298 K; optical path length = 0.02 cm.

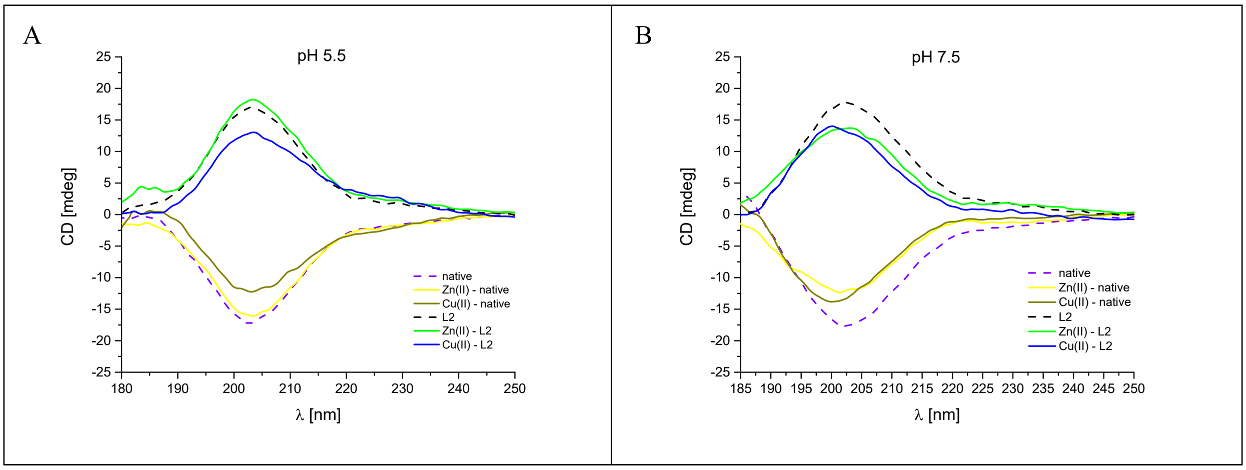

Figure S 9. Comparison of far-UV CD spectra for the peptide ligands: native (FPNPHQPPKHPDK), L1 (FPNPHQPPkhPDK) and L2 (fpnphqppkhpdk) and their Cu(II) and Zn(II) complexes, recorded at pH values: (A) 5.5 and (B) 7.5 in aqueous solution of 0.004 M $HClO_4$ with ionic strength I = 0.1 M ($NaClO_4$). [L] = 0.00035 M, molar ratio M:L = 0.9:1; T = 298 K; optical path length = 0.02 cm.

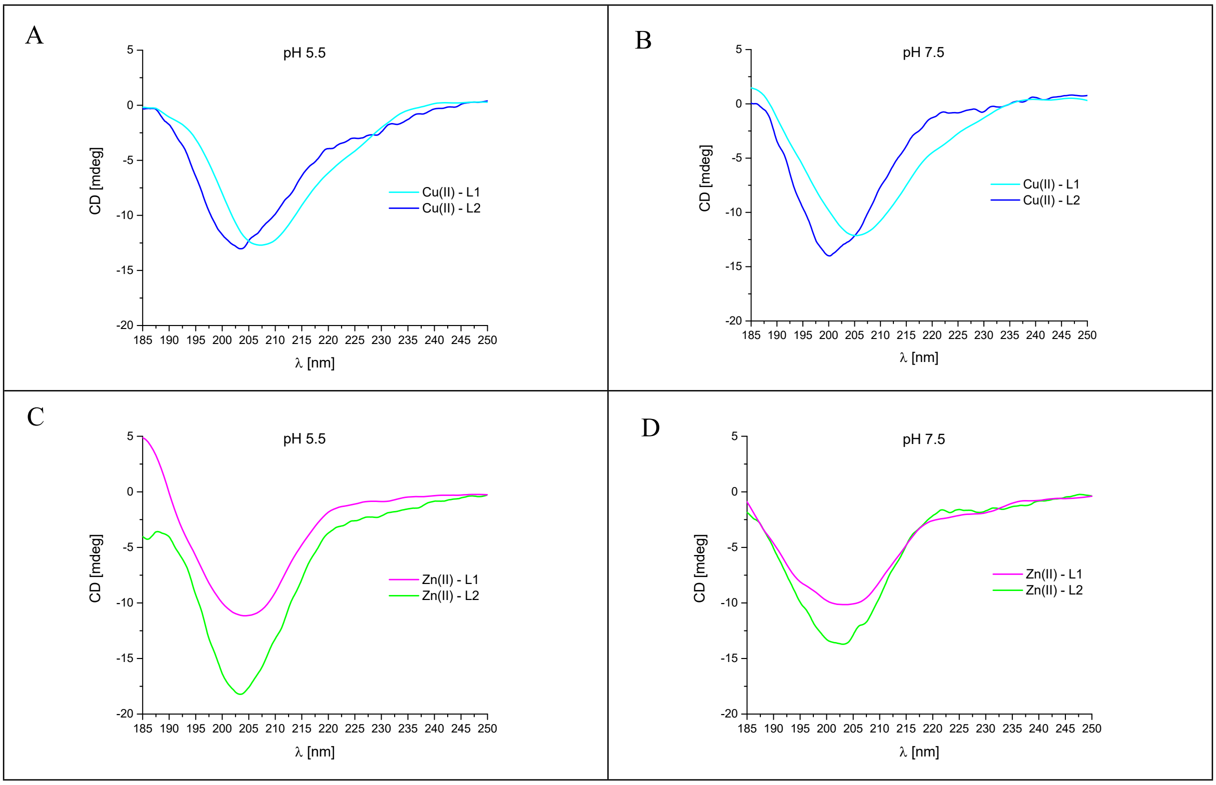

Figure S 10. Comparison of far-UV CD spectra for the peptide complexes: (A) Cu(II) - L1 and Cu(II) - L2 at pH 5.5, (B) Cu(II) - L1 and Cu(II) - L2 at pH 7.5, (C) Zn(II) - L1 and Zn(II) - L2 at pH 5.5 and (D) Zn(II) - L1 and Zn(II) - L2 at pH 7.5 in aqueous solution of 0.004 M $HClO_4$ with ionic strength I = 0.1 M ($NaClO_4$). [L] = 0.00035 M, molar ratio M:L = 0.9:1; T = 298 K; optical path length = 0.02 cm.

Table S 2. The antibacterial and anti-Candida activities of copper (Cu) and zinc (Zn) metal ions were assessed in vitro by determining their MIC (μg/mL). Antimicrobial tests were conducted in a 10 mM MES buffer at pH 5.5. n/d, not determined within the concentration range used in this study.

**Strain**	**Cu(II)**	**Zn(II)**
E. coli ATCC 25922	n/d	n/d

P. aeruginosa ATCC 15422	n/d	n/d
E. faecalis ATCC 29212	n/d	n/d
S. aureus ATCC 25923	n/d	n/d
S. mutans PCM 2502	n/d	n/d
S. sanguinis PMC 2335	n/d	n/d
C. albicans SC5314	n/d	n/d

Experiments were performed with copper (Cu(II)) and zinc (Zn(II)) ions at concentrations ranging from 0.3 to 38 µg/mL, corresponding to the metal ion concentrations present in the respective complexes.

Table S 3. The antibacterial and anti-Candida activities of copper (Cu) and zinc (Zn) metal ions were assessed in vitro by determining their MIC (µg/mL). Antimicrobial tests were conducted in a 10 mM MES buffer at pH 7.4. n/d, not determined within the concentration range used in this study.

**Strain**	**Cu(II)**	**Zn(II)**
E. coli ATCC 25922	n/d	n/d
P. aeruginosa ATCC 15422	n/d	n/d
E. faecalis ATCC 29212	n/d	n/d
S. aureus ATCC 25923	n/d	n/d
S. mutans PCM 2502	n/d	n/d
S. sanguinis PMC 2335	n/d	n/d
C. albicans SC5314	n/d	n/d

Experiments were performed with copper (Cu(II)) and zinc (Zn(II)) ions at concentrations ranging from 0.3 to 38 µg/mL, corresponding to the metal ion concentrations present in the respective complexes.

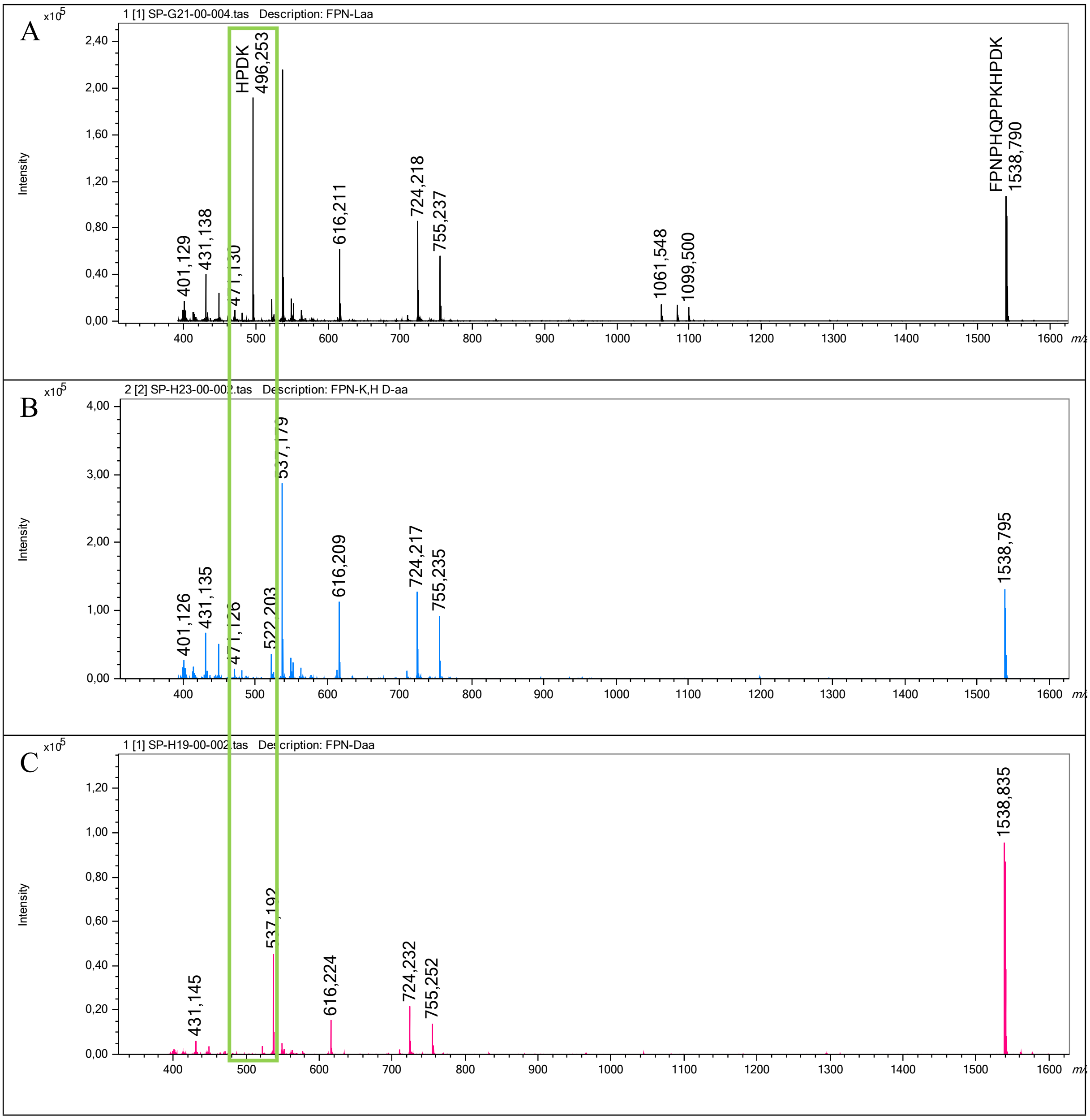

Figure S 11. MALDI-TOF MS spectra of (A) FPNPHQPPKHPDK (native peptide), (B) FPNPHQPPKhPDK (L2, 2 D-aa), and (C) fpnphqppkhpdk (L3, all D-aa) peptides after trypsin digestion. Each sample consists of peptide, phosphate buffer, and trypsin. The signal at m/z 496.253 corresponds to the HPDK fragment; this signal is absent in the peptidomimetics (B and C), indicating proteolytic resistance at the modified site in the peptide chain.

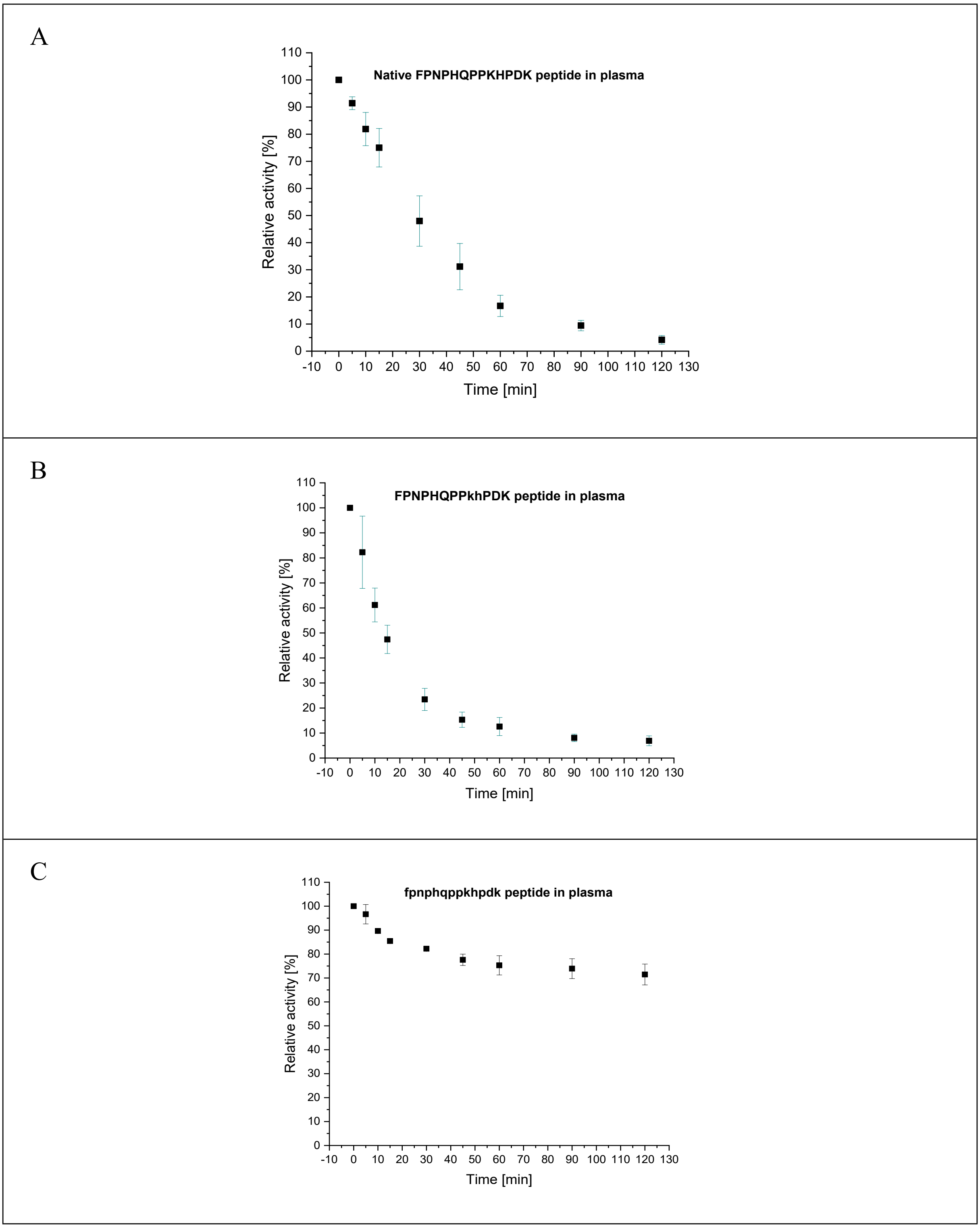

Figure S 12. Relative activity of the (A) FPNPHQPPKHPDK (native peptide), (B) FPNPHQPPKhPDK (L2, 2 D-aa), and (C) fpnphqppkhpdk (L3, all D-aa) fpnphqphkpdk peptide in plasma over time. Data are presented as mean values with error bars indicating variability between measurements. peptide in plasma over time. UV detection at λ=200 nm, [L] = 0.1 mM in aqueous solution of 0.01 M ammonium acetate buffer (pH 7.4); phenylalaninol (8 mM) was used as an internal standard, acetonitrile/water (13:87, v/v) with 0.1% TFA as the mobile phase, and reactions were quenched with 0.5 M $HClO_4$. Samples were incubated at 37 °C and analyzed using plasma from the same donor.

[1] J. Gawłowski, A. Ślusarczyk, K. Szarszoń, F. Zobi, T. Janek, J. Wątły, "Short but promising – how nature modulates the antimicrobial activity of proline-rich fragment of salivary MUC-7" Dalton Transactions 2025, DOI 10.1039/D5DT01418B.
